# Supplementary material for: Accuracy of emergency medical service telephone triage of need for an ambulance response in suspected COVID-19: an observational cohort study
Source: BMJ Open. 2022 May 16;12(5):e058628. doi: 10.1136/bmjopen-2021-058628 (PMC9114316; doi:10.1136/bmjopen-2021-058628)
Supplement: Supplementary data [file bmjopen-2021-058628supp001.pdf]

Supplementary Material 1: Summary of AMPDS pandemic Card 36 (Created by the authors) (full details available: https://cdn.emergencydispatch.org/iaed/pdf/resource-library/public%20protocols/pub-protocol%2036/NAE%20CC%2036.pdf)

Questions	Outcome
1) What is the most prominent complaint?	**Delta Category (immediate response)**<br>**Category 1/2 UK Ambulance service response**<br>1) INEFFECTIVE BREATHING with flu-like symptoms<br>2) DIFFICULTY SPEAKING BETWEEN BREATHS with flu-like symptoms<br>3) Not alert with flu-like symptoms<br>4) CHANGING COLOR with flu-like symptoms
If breathing a)Do they have difficulty speaking between breathes?<br>b) Describe their breathing<br>c)Did they have any flu symptoms prior to this?	
If Chest pain a) Are they 35 and over?<br>b) Have they had a heart attack or angina previously	
2) Are they completely alert?	
3) Are they changing colour?	
4) Are they having chills or sweats?	**Charlie Category (Ambulance when able)**<br>**Category 3/4 UK Ambulance service response**<br>1) Abnormal breathing with single flu-like symptom or Asthma/COPD<br>2) Abnormal breathing with multiple flu-like symptoms<br>3) Chest pain/discomfort ≥35 with single flu-like symptom<br>4) Chest pain/discomfort ≥35 with multiple flu-like symptoms<br>5) High Risk Factor
5) Are they vomiting?	
6)Do they have a new cough that recently started?	
7)Do they have a sore throat?	
8)Do they have unusual total body aches?	
9)Do they have a fever?	**Alpha Category (No ambulance dispatched)**<br>1) Chest pain/discomfort < 35 with single flu-like symptom<br>2) Chest pain/discomfort < 35 with multiple flu-like symptoms<br>3) Flu-like symptoms only
10)Do they have a runny or snotty nose?	
11)Do they have diarrhoea?	
12)Do they have a headache?	
13)Do they have any high-risk conditions?<br>• ≤ 5 years old (not COVID-19)<br>• ≥ 65 years old<br>• Blood disorders<br>• Diabetes<br>• Kidney and liver diseases/disorders<br>• Neurological diseases<br>• Pregnancy (up to 2 weeks after delivery)<br>• Sickle cell disease (sickle cell anaemia)<br>• Weakened immune system	

Marincowitz C, *et al. BMJ Open* 2022; 12:e058628. doi: 10.1136/bmjopen-2021-058628

Supplementary Material 2: Multi-variable model predicting false negatives (Created by the authors)

Population Characteristic	Level	Odds ratio (95% CI) N= 1215
**Age (Years)**	1-year increase	0.95 (0.94 to 0.97)
**Sex**	Female	1.89 (1.09 to 3.26)
**Comorbidity**	Cardiovascular Disease	0.73 (0.21 to 2.56)
	Chronic Resp. Disease	0.50 (0.23 to 1.09)
	Diabetes	1.19 (0.60 to 2.38)
	Hypertension	0.75 (0.39 to 1.46)
	Immunosuppression (including steroid use)	0.73 (0.35 to 1.53)
	Active Malignancy	0.12 (0.02 to 0.92)
	Obesity	Not included
	Renal Impairment	2.22 (0.71 to 6.96)
	Smoker	0.96 (0.52 to 1.80)
	Stroke	1.93 (0.42 to 8.80)
**Number of Drugs Used**	0	Reference
	1-5	0.98 (0.45 to 2.15)
	6-10	1.56 (0.55 to 4.44)
	11 or more	0.76 (0.08 to 7.42)
**Deprivation Index**	1-2	Reference
	3-4	0.98 (0.47 to 2.08)
	5-6	0.54 (0.21 to 1.36)
	7-8	1.37 (0.64 to 2.97)
	9-10	0.99 (0.38 to 2.59)

Supplementary Material 3: Multi-variable model predicting false positives

Population Characteristic	Level	Odds ratio (95% CI) N= 5, 615
**Age (Years)**	1-year increase	1.05 (1.04 to 1.05)
**Sex**	Female	1.31 (1.15 to 1.49)
**Comorbidity**	Cardiovascular Disease	1.38 (0.85 to 2.24)
	Chronic Resp. Disease	1.35 (1.13 to 1.60)
	Diabetes	0.85 (0.67 to 1.09)
	Hypertension	1.03 (0.84 to 1.26)
	Immunosuppression (including steroid use)	1.02 (0.82 to 1.26)
	Active Malignancy	1.25 (0.72 to 2.18)
	Renal Impairment	0.80 (0.44 to 1.44)
	Smoker	0.86 (0.75 to 0.99)
	Stroke	1.17 (0.59 to 2.31)
**Number of Drugs Used**	0	Reference
	1-5	0.99 (0.84 to 1.16)
	6-10	1.22 (0.90 to 1.66)
	11 or more	1.13 (0.62 to 2.06)
**Deprivation Index**	1-2	Reference
	3-4	1.21 (1.02 to 1.44)
	5-6	1.23 (1.01 to 1.48)
	7-8	1.29 (1.05 to 1.59)
	9-10	1.32 (1.04 to 1.69)
