# Supplementary material for: Accuracy of emergency medical service telephone triage of need for an ambulance response in suspected COVID-19: an observational cohort study
Source: BMJ Open. 2022 May 16;12(5):e058628. doi: 10.1136/bmjopen-2021-058628 (PMC9114316; doi:10.1136/bmjopen-2021-058628)
Supplement: Author's manuscript [file bmjopen-2021-058628.draft_revisions.pdf]

BMJ Open is committed to open peer review. As part of this commitment we make the peer review history of every article we publish publicly available.

When an article is published we post the peer reviewers' comments and the authors' responses online. We also post the versions of the paper that were used during peer review. These are the versions that the peer review comments apply to.

The versions of the paper that follow are the versions that were submitted during the peer review process. They are not the versions of record or the final published versions. They should not be cited or distributed as the published version of this manuscript.

BMJ Open is an open access journal and the full, final, typeset and author-corrected version of record of the manuscript is available on our site with no access controls, subscription charges or pay-per-view fees (http://bmjopen.bmj.com).

If you have any questions on BMJ Open's open peer review process please email
info.bmjopen@bmj.com

BMJ Open

Accuracy of emergency medical service telephone triage of need for an ambulance response in suspected COVID-19: An observational cohort study

Journal:	*BMJ Open*
Manuscript ID	bmjopen-2021-058628
Article Type:	Original research
Date Submitted by the Author:	29-Oct-2021
Complete List of Authors:	Marincowitz, Carl; The University of Sheffield, Centre for Urgent and Emergency Care Research<br>Stone, Tony; The University of Sheffield, Centre for Urgent and Emergency Care Research<br>Hasan, Madina ; The University of Sheffield, Centre for Urgent and Emergency Care Research<br>Campbell, Richard; The University of Sheffield, Centre for Urgent and Emergency Care Research<br>Bath, Peter; The University of Sheffield, Information School<br>Turner, Janette; The University of Sheffield, Centre for Urgent and Emergency Care Research<br>Pilbery, Richard; Yorkshire Ambulance Service NHS Trust, Research and Development<br>Thomas, Benjamin; The University of Sheffield, Clinical Trials Research Unit<br>Sutton, Laura ; The University of Sheffield, Clinical Trials Research Unit<br>Bell, Fiona; Yorkshire Ambulance Service NHS Trust, Research and Development<br>Biggs, Katie; The University of Sheffield, Clinical Trials Research Unit<br>Hopfgartner, Frank; The University of Sheffield, Information School<br>Mazumdar, Suvodeep; The University of Sheffield, Information School<br>Petrie, Jennifer; The University of Sheffield, Clinical Trials Research Unit<br>Goodacre, Steve; The University of Sheffield, Centre for Urgent and Emergency Care Research
Keywords:	COVID-19, Risk management < HEALTH SERVICES ADMINISTRATION & MANAGEMENT, ACCIDENT & EMERGENCY MEDICINE

SCHOLARONE™
Manuscripts

I, the Submitting Author has the right to grant and does grant on behalf of all authors of the Work (as defined in the below author licence), an exclusive licence and/or a non-exclusive licence for contributions from authors who are: i) UK Crown employees; ii) where BMJ has agreed a CC-BY licence shall apply, and/or iii) in accordance with the terms applicable for US Federal Government officers or employees acting as part of their official duties; on a worldwide, perpetual, irrevocable, royalty-free basis to BMJ Publishing Group Ltd ("BMJ") its licensees and where the relevant Journal is co-owned by BMJ to the co-owners of the Journal, to publish the Work in this journal and any other BMJ products and to exploit all rights, as set out in our licence.

The Submitting Author accepts and understands that any supply made under these terms is made by BMJ to the Submitting Author unless you are acting as an employee on behalf of your employer or a postgraduate student of an affiliated institution which is paying any applicable article publishing charge ("APC") for Open Access articles. Where the Submitting Author wishes to make the Work available on an Open Access basis (and intends to pay the relevant APC), the terms of reuse of such Open Access shall be governed by a Creative Commons licence – details of these licences and which Creative Commons licence will apply to this Work are set out in our licence referred to above.

Other than as permitted in any relevant BMJ Author's Self Archiving Policies, I confirm this Work has not been accepted for publication elsewhere, is not being considered for publication elsewhere and does not duplicate material already published. I confirm all authors consent to publication of this Work and authorise the granting of this licence.

For peer review only

Accuracy of emergency medical service telephone triage of need for an ambulance response in suspected COVID-19: An observational cohort study

Carl Marincowitz[1] NIHR Clinical Lecturer Emergency Medicine

Tony Stone[1] Senior Data Manager

Madina Hasan[1] Research Associate

Richard Campbell[1] Data Specialist

Peter A. Bath[1,2] Professor of Health Informatics and Health Data Analytics

Janette Turner[1] Honorary Senior Research Fellow

Richard Pilbery[3] Research paramedic

Benjamin Thomas[4] Study Manager

Laura Sutton[4] Research Associate

Fiona Bell[3] Head of Research

Katie Biggs[4] Research Fellow/Assistant CTRU Director

Frank Hopfgartner[2] Senior Lecturer in Data Science

Suvodeep Mazumdar[2] Lecturer in Data Analytics

Jennifer Petrie[4] Trial Manager

Steve Goodacre[1] Professor of Emergency Medicine

1. Centre for Urgent and Emergency Care Research (CURE), Health Services Research School of Health and Related Research, University of Sheffield, Regent Court, 30 Regent Street, Sheffield, S1 4DA, UK, Fax: +44 (0)114 222 0749 Tel: (+44) (0)114 222 4345, Email: C.Marincowitz@Sheffield.ac.uk
2. Information School, University of Sheffield, Regent Court, 211 Portobello St, Sheffield S1 4DP, UK
3. Yorkshire Ambulance Service NHS Trust, Trust HQ, Springhill 2, Brindley Way, Wakefield, United Kingdom, WF2 0XQ
4. Clinical Trials Research Unit (CTRU), Health Services Research School of Health and Related Research, University of Sheffield, Regent Court, 30 Regent Street, Sheffield, S1 4DA, UK, Fax: +44 (0)114 222 0749 Tel: (+44) (0)114 222 4345,

Correspondence to Dr Benjamin Thomas at b.d.thomas@sheffield.ac.uk

For peer review only - http://bmjopen.bmj.com/site/about/guidelines.xhtml

Abstract

Objective: To assess accuracy of Emergency Medical Service (EMS) telephone triage in identifying patients who need an EMS response and identify factors which affect triage accuracy.

Design: Observational cohort study

Setting: Emergency telephone triage provided by Yorkshire Ambulance Service NHS Trust (YAS).

Participants: 12, 653 adults who contacted EMS telephone triage services provided by YAS between the 2ndApril 2020 and 29th June 2020 assessed by COVID-19 telephone triage pathways were linked to Office for National Statistics death registrations and health care data collected by NHS Digital.

Outcome: Accuracy of call handler decision to dispatch an ambulance was assessed in terms of death or need for organ support at 30 days from first contact with the telephone triage service.

Results: Callers had an 11.1% of the primary outcome. Using the triage pathway, 16% of callers did not receive an emergency response and they had a 3.5% risk of death or organ support. Ambulances were dispatched to 4, 230 callers (33.4%) who were not conveyed to hospital and did not deteriorate. Multivariable modelling found older age and presence of pre-existing respiratory disease were significant predictors of false positive triage.

Conclusion: Telephone triage avoided an emergency response for 16% of callers, of whom 3.5% died or needed organ support by 30 days. Telephone triage can reduce ambulance responses but with the cost of a small proportion of patients who do not receive an initial emergency response deteriorating.

Strengths and limitations of this study

- Despite concerns regarding accuracy in identifying need for emergency treatment, this is one of the first evaluations of EMS telephone triage protocols for suspected COVID-19.
- Use of ambulance data linked to nationally collected death registrations and routinely collected healthcare data provided robust outcome data for all included callers.
- Use of routine data limited assessment of factors which affect triage accuracy to those which are routinely collected.
- The evaluation is of single UK amulance service's implementation of EMS telephone triage protocols for suspected COVID-19.

Word Count: 3,120

For peer review only - http://bmjopen.bmj.com/site/about/guidelines.xhtml

Background

During the COVID-19 pandemic, emergency medical service (EMS) call volumes have been volatile in the UK, Europe and North America. Some ambulance services in the UK reported up to three times the expected number of EMS calls during the first and second waves of the pandemic, an increase also observed in other parts of Europe.[1-4] Other EMS providers in the UK and North America observed initial decreases in call volumes.[5, 6] Surges in demand in the second wave of the pandemic led to some ambulance services in the UK declaring major incidents and warning of care being compromised by overwhelming demand.[7, 8]

EMS call handlers in the UK are typically trained non-clinical staff who use either the Advanced Medical Priority Dispatch System (AMPDS) or NHS Pathways to triage what emergency care response is needed to calls.[4] On the 2nd April 2020, six English NHS ambulance services using AMPDS introduced a specific protocol for callers with suspected COVID-19 using the pandemic Card 36.[9, 10] Structured questions (summarised in Supplementary Material 1) were used to triage urgency of EMS response into three levels: Delta (highest priority requiring immediate response), Charlie (medium priority requiring ambulance attendance when able) and Alpha (no ambulance dispatched). The AMPDS Card 36 was implemented by UK ambulance services in different ways, with some symptoms and selected high risk-groups receiving a higher priority response than normal, with alterations to the card implemented in the following months.[4]

There is evidence that changes in EMS practice due to the pandemic and delays in the emergency assessment of patients with COVID-19 may have contributed to avoidable deaths in the North West of England.[11] Despite the need for EMS telephone triage to balance ambulance responses to patients requiring life-saving interventions against an anticipation of overwhelming demand during the pandemic, there has been no previous evaluation of the accuracy of the clinical risk-assessment performed for patients with suspected COVID-19.

Our study aimed to:

1) Assess how accurately EMS telephone triage identified those likely to suffer an adverse outcome and so need an emergency ambulance response.
2) Identify any factors that may have affected the accuracy of EMS telephone triage.

Methods

Study Design and Setting

For peer review only - http://bmjopen.bmj.com/site/about/guidelines.xhtml

This observational cohort study used linked routinely collected EMS telephone call centre data from Yorkshire Ambulance Service NHS Trust (YAS) to assess the accuracy of clinical triage of patients with suspected COVID-19.

Emergency services provided by YAS covers a region in the north of England of approximately 6,000 square miles and with a population of 5.3 million. In 2020/2021, YAS received more than 1,000,000 emergency (999) calls.

Data Sources and linkage

Yorkshire Ambulance Service provided a dataset of all 999 calls, triaged using a Card 36 pandemic triage pathway for patients with suspected COVID-19, received between the 2ndApril and 29th June 2020. The dataset consisted of patient identifiers, demographic data, call details and the outcome of the call (including whether an ambulance was dispatched) extracted from routinely collected electronic call records.

Health and social care data relating to the population in England within the UK National Health Service (NHS) are managed by NHS Digital. We provided patient identifiers to NHS Digital to trace patients in our cohort and supply additional individual-level demographic, co-morbidity and outcome data. NHS Digital identified records in their collections belonging to patients in our cohort, and provided data on patient demographics, limited COVID-related general practice (GP) records, emergency department attendances, hospital inpatient admissions, critical care periods, and death registrations from the UK Office for National Statistics (ONS).

YAS and NHS Digital removed records where patients indicated that they did not wish their data to be used for research purposes, via the NHS data opt-out service.[12] The study team also excluded patients who had opted out of any part of the wider PRIEST study[2], of which this evaluation forms a part, and those with inconsistent records (e.g., multiple deaths recorded or death before latest activity). Patient identifiers across all datasets were replaced with a consistent pseudo-identifier to enable the identification and linkage of records belonging to the same patient across all datasets but without revealing any patient identifiers. Calls which originated from other health care services (i.e., where a decision that ambulance dispatch was required had already been made) were excluded from the study population.

Inclusion criteria

Our final cohort consisted of all adult (aged 16 years and over) callers at time of first (index) EMS 999 call between 2nd April and 29th June 2020 assessed using the suspected COVID-19 Card 36 triage pathway, who were successfully traced by NHS Digital.

For peer review only - http://bmjopen.bmj.com/site/about/guidelines.xhtml

Outcome

The primary outcome was death, renal, respiratory, or cardiovascular organ support (identified from death registration and critical care data) at 30 days from index contact.

The secondary outcomes were hospital conveyance following ambulance dispatch (transfer to ED or inpatient setting) and inpatient admission (recoded inpatient dataset) 30 days from index contact.

Patient Characteristics

Consistent with methods used to estimate the Charlson comorbidity index from the available routine data, comorbidities were included if recorded within 12 months before the index EMS call.[13, 14] In a similar way, only immunosuppressant drug prescriptions documented in GP records within 30 days before the index contact, contributed to the immunosuppression co-morbidity variable. Pregnancy status was based on GP records recorded in the previous 9 months. Frailty in patients older than 65 years was derived from the latest recorded Clinical Frailty Scale (CFS) score (if recorded) in the electronic GP records prior to index attendance.[15] Patients under the age of 65 years were not given a CFS score since it is not validated in this age group. However, in multivariable analysis, patients under the age of 65 were assumed to have a functional level equivalent to a mild frailty category (CFS 1-3).

Analysis

We conducted a descriptive analysis of caller demographics, co-morbidities and call disposition. The proportion of callers who experienced the primary and secondary outcome was estimated. To assess the accuracy of EMS telephone triage in identifying clinical outcomes requiring an emergency response, the call disposition of the index contact was divided into the binary classification of either: ambulance dispatched (Delta or Charlie priority); or other call outcome (Alpha priority) (Supplementary Material 1). We assessed the accuracy of the binary triage classification (ambulance dispatched versus no ambulance dispatched) in terms of sensitivity, specificity, positive predictive value (PPV) and negative predictive value (NPV) for the primary and secondary outcomes with 95% confidence intervals (CI).

Patient characteristics of false negatives (those who experienced the primary outcome and no ambulance was dispatched) and true positives (ambulance dispatched who experienced the primary outcome) were compared. In patients with the adverse outcome multi-variable logistic regression was used to identify patient characteristics associated with false negative triage. We also compared

For peer review only - http://bmjopen.bmj.com/site/about/guidelines.xhtml

the characteristics of false positives (ambulance dispatched and not conveyed to hospital) and true negatives (no ambulance dispatched) among those who did not experience the primary composite outcome. We used multivariable logistic regression to identify factors which predicted false positive EMS triage. Obesity was excluded from multivariable analysis due to an observed implausible protective association with the primary outcome, which we believe to be an artefact of how these data were collected and recorded in the electronic GP data set. Ethnicity and frailty were also excluded from multivariable analysis due to the high proportion of missing data. All analyses were completed using STATA 16 (StataCorp. 2019. *Stata Statistical Software: Release 16*. College Station, TX: StataCorp LLC).

Ethical Approval

The North West—Haydock Research Ethics Committee gave a favourable opinion on the PAINTED study on 25 June 2012 (reference 12/NW/0303) and on the updated PRIEST study on 23rd March 2020, including the analyses presented here. The Confidentiality Advisory Group of the NHS Health Research Authority granted approval to collect data without patient consent in line with Section 251 of the National Health Service Act 2006. Access to data collected by NHS Digital was recommended for approval by its Independent Group Advising on the Release of Data (IGARD) on 11th September 2021, having received additional recommendation for approval for access to GP records from the Profession Advisory Group (PAG) on 19th August 2021.

Patient Public Involvement

The Sheffield Emergency Care Forum (SECF) is a public representative group interested in emergency care research.[16] Members of SECF advised on the development of the PRIEST study and two members joined the Study Steering Committee. Patients were not involved in the conduct of the study.

Results

All totals (including outcome) presented are rounded to the nearest 5, with small numbers suppressed to comply with NHS Digital data disclosure guidance.[17]

Study population

For peer review only - http://bmjopen.bmj.com/site/about/guidelines.xhtml

Figure 1 and Table 1 summarise study cohort derivation and the characteristics of the 12, 653 included individual callers. In total, 1,405 callers (11.1%, 95% CI: 10.5 to 11.7%) experienced the primary outcome (death or organ support) within 30 days following the index EMS 999 call. An ambulance was dispatched to 10,650 (84.2%) of callers. In our study cohort, 6,070 patients (48%. 95%: 47.1 to 48.9%) were conveyed to hospital (ED or directly to an inpatient setting) and 5,735 (45.3%, 95% CI: 44.5 to 46.2%) were admitted as hospital inpatients within 30 days of index contact.

The median age of the whole cohort was 66 (IQR = 46-81), the cohort had an almost equal proportion of males (49.5%) and females (50.5%) and had high rates of comorbidity (chronic respiratory disease 29.5%, diabetes 16.6% and hypertension 35.9%).

Table 1: Population characteristics

Population Characteristic	Level	Whole Population N=12, 653	Adverse Outcome (30 days) N=1,405
**Age (Years)**	Median (IQR)*<br>Mean	66 (46-81)<br>62.3	80 (67-87)<br>70
**Sex (N, %)**	Male	6,260 (49.5%)	795 (56.5%)
**Comorbidity (N, %)**	Cardiovascular Disease	810 (6.4%)	120 (8.6%)
	Chronic Resp. Disease	3,730 (29.5%)	410 (29.4%)
	Diabetes	2,095 (16.5%)	325 (23.1%)
	Hypertension	4,545 (35.9%)	705 (50.4%)
	Immunosuppression (including steroid use)	2, 540 (20.1%)	360 (25.8%)
	Active Malignancy	595 (4.7%)	160 (11.5%)
	Obesity	1,960 (15.5%)	100 (7.3%)
	Pregnant	135 (1.1%)	*
	Renal Impairment	365 (2.9%)	60 (4.3%)
	Stroke	285 (2.2%)	45 (3.1%)
**Social (N, %)**	Smoker	4, 810 (38.0%)	550 (39.3%)
**Number of Prescribed Drugs Used (N, %)**	0	3, 115 (24.6%)	175 (12.4%)
	1-5	6, 015 (47.5%)	700 (49.8%)
	6-10	3, 030 (23.9%)	465 (33.1%)
	11 or more	490 (3.9%)	65 (4.7%)
**Clinical Frailty Scale (N, %)**	Unknown	4,560 (36.0%)	685 (48.9%)
	Aged<65	6,105 (48.2%)	305 (21.6%)

For peer review only - http://bmjopen.bmj.com/site/about/guidelines.xhtml

	1-3	165 (1.3%)	15 (1.0%)
	3-6	670 (5.3%)	85 (6.0%)
	6-9	1,155 (9.1%)	315 (22.5%)
**Ethnicity (N, %)**	Asian or Asian British	750 (5.9%)	5 (3.6%)
	Black or Black British	190 (1.5%)	15 (0.9%)
	Mixed	110 (0.9%)	*
	Other Ethnic Groups	160 (1.3%)	*
	White	9,020 (71.3%)	950 (67.8%)
	Unknown	2,425 (19.2%)	375 (26.6%)
**Deprivation Index (N, %)**	Unknown	1,250 (9.9%)	190 (13.5%)
	1-2	4,600 (40.3%)	440 (36.4%)
	3-4	2,075 (18.2%)	220 (18%)
	5-6	1,880 (16.5%)	220 (18%)
	7-8	1,695 (14.9%)	210 (17.2%)
	9-10	1,160 (10.2%)	130 (10.6%)
**Ambulance Dispatched (N, %)**	Ambulance	10,650 (84.2%)	1,335 (95%)
	No Ambulance	2,000 (15.8%)	70 (5%)
**Outcome (N, %)**	Death	1,155 (9.1%)	1,155 (82.4%)
	Deaths due to COVID (including after 30 days)	690 (5.5%)	530 (37.8%)
	Organ support (within 30 days)	335 (2.6%)	335 (23.8%)
**Hospitalisation (N, %)**	ED Attendance	6,945 (54.9%)	970 (69.1%)
	Inpatient admission	5,735 (45.3%)	1,085 (77.3%)
**Confirmed Hospital Diagnosis of COVID (N, %)****	In ED or as inpatient at 30 days	1,895 (15%)	595 (42.3%)
**Time to Primary Outcome from index contact- up to and including (N, %)**	72 hours	475 (3.8%)	475 (33.6%)
	7 days	780 (6.1%)	780 (55.5%)

*Interquartile Range (IQR)

**Unrestricted community testing for suspected COVID-19 was only available from the 18/05/2020. Confirmed diagnosis is based upon inpatient PCR testing or clinical diagnosis in hospital

Accuracy of telephone triage of ambulance dispatch

Table 2 shows the accuracy of the EMS telephone triage decision to dispatch an ambulance for the composite primary outcome indicating need for emergency intervention. Decision to dispatch an ambulance achieved a sensitivity of 95% (95% CI:93.7 to 96.1%) to the primary outcome. If advised

For peer review only - http://bmjopen.bmj.com/site/about/guidelines.xhtml

to self-care/non-urgent clinical assessment, the chance of experiencing an adverse outcome was approximately 3.5% (NPV: 96.5%, 95% CI: 95.6 to 97.2%). The high sensitivity was achieved at the cost of specificity (17.2% 95% CI: 16.5% to 17.9%). Among patients for whom an ambulance was dispatched, the risk of serious adverse outcomes was 12.5% (95% CI: 11.9 to 13.2%), transfer to hospital was 57% (95% CI: 56 to 58%) and hospital admission 49.3% (48.3 to 50.2%).

Table 2: Performance of decision to dispatch ambulance for

i) composite primary outcome (death or organ support)

Adverse outcome 30 days (11.1%, 10.5-11.7%)			
N= 12,653	**Adverse Outcome**	**No Adverse Outcome**	
Ambulance dispatched	1,335	9,320	Sensitivity 95% (93.7-96.1%)<br>Positive Predictive Value 12.5% (11.9 – 13.2%)
No Ambulance dispatched	70	1,930	Specificity 17.2% (16.5% -17.9%)<br>Negative Predictive Value 96.5% (95.6 - 97.2%)

Prediction of false negative or false positive ambulance dispatch

Table 3 compares the characteristics of patients who experienced the primary outcome, and either did (true positives) or did not (false negatives) have an ambulance dispatched on index call. In both groups, over 50% of people experienced the primary adverse outcome within seven days of first contact and around 33% of patients experienced the adverse outcome within 72 hours of index assessment. Multivariable modelling (Supplementary Material 2) showed that female sex (OR 1.89, 95% CI 1.09 to 3.26) was associated with increased risk and, increasing age (OR 0.95, 95% CI 0.94 to 0.97) and malignancy (OR 0.12, 95% CI 0.02 to 0.92) reduced risk, of false negative triage.

Table 3: False negatives (No ambulance dispatch and adverse outcome) compared to true positives (ambulance dispatch and adverse outcome)

**Population Characteristic**	**Level**	**False Negatives (30 days) N= 70**	**True Positives (30 days) N= 1,335**
**Age (Years)**	Median (IQR)<br>Mean	61.5 (51-80)<br>64	80 (68-87)<br>76.7
**Sex (N, %)**	Male	35<br>(51.4%)	760<br>(56.8%)

For peer review only - http://bmjopen.bmj.com/site/about/guidelines.xhtml

**Comorbidity (N, %)**	Cardiovascular Disease	*	115 (8.8%)
	Chronic Resp. Disease	15 (20%)	400 (29.9%)
	Diabetes	20 (25.7%)	305 (23%)
	Hypertension	25 (34.3%)	685 (51.2%)
	Immunosuppression (including steroid use)	10 (17.1%)	350 (26.3%)
	Active Malignancy	*	160 (11.9%)
	Obesity	10 (12.9%)	95 (7%)
	Pregnant	*	*
	Renal Impairment	*	55 (4.2%)
	Stroke	*	40 (3.2%)
**Social (N, %)**	Smoker	25 (32.9%)	530 (39.6%)
**Number of Drugs Used (N, %)**	0	20 (25.7%)	155 (11.7%)
	1-5	30 (42.9%)	670 (50.2%)
	6-10	20 (28.6%)	445 (33.3%)
	11 or more	*	65 (4.8%)
**Clinical Frailty Scale (N, %)**	Unknown	60 (84.3%)	665 (49.8%)
	Aged<65	*	260 (19.7%)
	1-3	*	15 (1%)
	3-6	*	80 (6.2%)
	6-9	*	310 (23.4%)
**Ethnicity (N, %)**	Asian or Asian British	*	45 (3.5%)
	Black or Black British	*	15 (1%)
	Mixed	*	*
	Other Ethnic Groups	*	10 (0.7%)
	White	45 (61.4%)	910 (68.1%)
	Unknown	20 (28.6%)	355 (26.5%)
**Deprivation Index (N, %)**	Unknown	*	185 (13.7%)
	1-2	30 (46%)	410 (35.8%)
	3-4	10 (17.4%)	205 (17.9%)
	5-6	*	210 (18.4%)
	7-8	10 (17.4%)	200 (17.2%)
	9-10	*	120 (10.6%)
**Outcome (N, %)**	Death	45 (65.7%)	1,110 (83.3%)
	Deaths due to COVID (including after 30 days)	20 (27.2%)	520 (39%)
	Organ support (within 30 days)	35 (47.1%)	300 (22.6%)
**Hospitalisation (N, %)**	ED attendance	45 (65.7%)	925 (69.2%)
	Inpatient admission	50 (72.9%)	1,035 (77.6%)
**Confirmed Hospital Diagnosis of COVID (N, %)****	In ED or as inpatient at 30 days	25 (34.3%)	570 (42.7%)
**Time to Primary Outcome from index contact- up to and including (N, %)**	72 hours	25 (37.1%)	445 (33.5%)
	7 days	45 (62.8%)	730 (54.9%)

For peer review only - http://bmjopen.bmj.com/site/about/guidelines.xhtml

Table 4 compares the characteristics of patients without the primary outcome for whom an ambulance was dispatched and the patient was not transferred to hospital (False Positives), or no ambulance was dispatched (True Negatives). 33.4% of the cohort were false positives and Supplementary Material 3 presents the results of multivariable modelling to identify factors associated with false postivie triage. Risk of false positive was strongly associated with chronic respiratory disease (OR 1.35 95% CI 1.13 to 1.60) and reduced in smokers (OR 0.86, 95% CI 0.75 to 0.99). Increasing age, deprivation and female sex were also associated with risk of false positive triage.

Table 4: False positive (Ambulance dispatched, not conveyed to hospital and no adverse outcome) compared to true negatives

Population Characteristic	Level	False Positive (30 days) N= 4,230	True Negative (30 days) N= 1,930
**Age (Years)**	Median (IQR)<br>Mean	66 (46-81)<br>62.9	37 (28-53)<br>41.6
**Sex (N, %)**	Male	1,920 (45.4%)	1000 (51.7%)
**Comorbidity (N, %)**	Cardiovascular Disease	235 (5.5%)	30 (1.6%)
	Chronic Resp. Disease	1,280 (30.3%)	380 (19.5%)
	Diabetes	600 (14.2%)	155 (7.8%
	Hypertension	1,480 (35%)	255(13.1%)
	Immunosuppression (including steroid use)	825 (19.5%)	180 (9.4%)
	Active Malignancy	140 (3.3%)	20 (0.9%)
	Obesity	700 (16.5%)	260 (13.4%)
	Pregnant	50 (1.2%)	40 (2.2%)
	Renal Impairment	110 (2.6%)	15 (0.8%)
	Stroke	90 (2.1%)	15 (0.7%)
**Smoking Status (N, %)**	Smoker	1,570 (37.1%)	640 (33.2%)
**Number of Drugs Used (N, %)**	0	1,040 (24.6%)	955 (49.3%)
	1-5	2,075 (49%)	805 (41.7%)
	6-10	970 (22.9%)	150 (7.6%)
	11 or more	150 (3.5%)	25 (1.4%)
**Clinical Frailty Scale (N, %)**	Unknown	1,520 (35.9%)	150 (7.6%)
	Aged<65	2,030 (48%)	1,735 (89.8%)
	1-3	55 (1.3%)	*
	3-6	245 (5.7%)	25 (1.2%)
	6-9	385 (9.1%)	25 (1.2%)
**Ethnicity (N, %)**	Asian or Asian British	250 (5.9%)	180 (9.2%)
	Black or Black British	60 (1.4%)	50 (2.5%)
	Mixed	35 (0.9%)	30 (1.4%)

For peer review only - http://bmjopen.bmj.com/site/about/guidelines.xhtml

	Other Ethnic Groups	50 (1.2%)	65 (3.3%)
	White	3,080 (72.7%)	1,210 (62.7%)
	Unknown	760 (17.9%)	405 (20.9%)
**Deprivation Index (N, %)**	Unknown	420 (10%)	130 (6.6%)
	1-2	1,470 (38.6%)	925 (51.3%)
	3-4	720 (18.9%)	320 (17.7%)
	5-6	655 (17.2%)	245 (13.5%)
	7-8	560 (14.7%)	190 (10.5%)
	9-10	405 (10.6%)	125 (6.9%)

Discussion

Summary

Our study showed that callers with suspected COVID-19 who made an EMS call had a high rate of adverse outcomes (11.1%, 95% CI: 10.5 to 11.7%). This is around four times greater than the adverse outcome rate in callers who contacted the NHS 111 telephone service and half that seen in an ED population with suspected COVID-19.[18, 19] Ambulances were dispatched to the majority of callers (84.2%) and the decision to dispatch an ambulance provided good, but not excellent, prediction for adverse outcomes (sensitivity 95%, 95% CI: 93.7 to 96.1%). Callers for whom an ambulance was not dispatched had a 3.5% (NPP 96.5% 95% CI: 95.6 to 97.2%) risk of the primary composite adverse outcome. Our evaluation cannot account for instances where ambulance dispatch was not clinically appropriate despite a high-risk of significant adverse outcomes (e.g. transfer of care to community services for palliative care).

The cost of the high sensitivity achieved by dispatching ambulances to 84% of callers was a low specificity to the primary outcome (17.2% 95% CI: 16.5% to 17.9%). In total, 33.4% of the cohort had an ambulance dispatched, and were not subsequently conveyed to hospital and nor did they experience the primary outcome. We used multivariable analysis to identify predictors of false negative and false positive triage. The findings need cautious interpretation, given the limited information available during telephone triage, but suggest that some co-morbidities (such as chronic respiratory disease) and increasing age may be over-estimated as predictors of adverse outcome.

Strengths and limitations

Although specific EMS telephone triage protocols have been introduced for patients with suspected COVID-19, this appears to be the first evaluation of triage accuracy.[4] Our study used a large cohort of patients identified from routinely collected EMS records and linked this to nationally collected, patient-level healthcare data to provide robust clinical outcomes. We have assessed performance in a cohort of patients with suspected infection which, in the absence of accurate universally available

For peer review only - http://bmjopen.bmj.com/site/about/guidelines.xhtml

rapid COVID-19 diagnostic tests, reflects the population who must be clinically triaged by urgent and emergency care services.

We have evaluated the performance EMS telephone triage for patients with suspected COVID-19 implemented by the Yorkshire Ambulance Service NHS Trust. Although use of the Card 36 protocol was recommended nationally, there was variation in implementation between different ambulance services.[4] Our study used data from the first wave of the pandemic and it was not until later waves that some ambulance services came under significant pressures due to increased demand.[1] Differences in EMS telephone triage demand and population characteristics of callers with suspected COVID-19 in later waves of the pandemic may affect the estimated accuracy of EMS decision to dispatch an ambulance. The implementation of a senior clinical support model within the call centre, and the influence of this secondary triage both of incoming calls and to support on-scene ambulance decision making was not quantified in this study. It was not possible to identify within the dataset which of the cohort were reviewed by a senior clinician at the initial call.

Our study used routinely collected data and consequently there were high rates of missing data for some variables, such as ethnicity and frailty. This prevented inclusion of these and non-routinely collected variables in analysis. We have assumed that if comorbidities or medication use was not recorded, they were not present. The mechanism of how data are collected and recorded in the routine data sets used means that there may be bias in the classification of patients. We have previously identified this for the obesity variable where the estimated prevalence of obesity in our cohort is 15% (half that reported in the national health survey) and an implausible protective association with adverse outcomes was observed.[18, 20] As weight is not comprehensively and consistently measured by GPs, estimated statistical effects may reflect unknown characteristics associated with a measurement being taken, rather than the variable itself.

Implications

EMS telephone triage of ambulance dispatch achieved a higher sensitivity for adverse outcomes than triage methods used for patient acuity in the ED and NHS-111 COVID-19 assessment pathways.[21, 22] Our cohort had a baseline risk of 11.1% of the primary outcome and serious adverse outcomes. The EMS telephone triage system selected those with a lower, 3.5% risk of the primary outcome, not to have an ambulance dispatched. Given the high baseline risk, an alternative would have been to dispatch an ambulance to every caller. This would have led to around 2000 more ambulance being dispatched within the time-period (an 18.8% increase). However, even using EMS telephone triage of ambulance dispatch, around a third of ambulances were dispatched to callers who were not subsequently conveyed to hospital and did not experience the primary outcome. In

For peer review only - http://bmjopen.bmj.com/site/about/guidelines.xhtml

later waves of the pandemic, EMS providers in the UK experienced significant increases in demand with several ambulance services declaring major incidents.[8, 23, 24] The accuracy of triage observed in our cohort may be the best that realistically can be achieved given the limited information available with telephone triage. However, any measures which can increase accuracy of EMS telephone triage, especially in terms of increasing specificity to adverse outcomes, without reducing sensitivity, are greatly needed.

Our exploratory multivariable analysis indicates that older age and presence of pre-existing respiratory disease were associated with a higher rate of false positive triage among those who did not have an adverse event, and therefore may be overestimated in the triage process as predictors of adverse outcomes. Being aged 65 years or over is included as a specific high-risk factor in the Card 36 pandemic triage tool.[25] Use of a higher age threshold, or age in conjunction with variables related to performance status, could improve the specificity of EMS ambulance dispatch to significant adverse outcomes.[26] Vaccination against COVID-19 or previous infection may also act to reduce the risk of serious adverse outcomes in suspected infection and these factors are not included in Card 36 assessment.[27]

Limited information is available during EMS telephone triage determining whether ambulance dispatch is required. The Card 36 pandemic triage tool used for this decision making in patients with suspected COVID-19 is consensus based. Empirical research assessing the predictive effect of all available variables is required if accuracy of triage, especially in terms of current over-triage, is to be improved. Given the limitations of the available routine data we have identified in this study, this is likely to require robust prospective data collection. The current high levels of demand that ambulance services are experiencing mean that even small gains in specificity which do not compromise sensitivity could have a large positive effect.

Conclusion

EMS telephone triage of need for ambulance dispatch in the first wave of the pandemic identified a population to whom ambulances were not dispatched, with a 3.5% risk of serious adverse outcomes in a population of callers with a baseline risk of 11.1%. The lower risk population constituted 16% of the cohort and around 2000 more ambulances would have been dispatched if these callers received an EMS response. As the pandemic has developed into later waves, ambulance services in the UK have come under significant sustained pressure due to increased demand. Research to improve the accuracy of EMS telephone triage, especially in terms of specificity, is needed. Due to the limitations of available routinely collected data this is likely to require robust prospective data collection.

For peer review only - http://bmjopen.bmj.com/site/about/guidelines.xhtml

For peer review only

Competing Interests

No competing financial interests exist.

Funding

CM is a National Institute for Health Research (NIHR) Clinical Lecturer in Emergency Medicine (Grant Number Not Applicable/NA). This publication presents independent research funded by the National Institute for Health Research and University of Sheffield. The views expressed are those of the author(s) and not necessarily those of the University of Sheffield, the NHS, the NIHR or the Department of Health and Social Care

Authors' contributions

The idea for the study was conceived by SG, JT, TS, FB, PB and CM. Data processing and linkage was completed by TS and RC. The analyses were completed by CM and MH with specialist statistical advice from SG, JT, LS and PB. All authors contributed to interpretation of results, read and approved the final manuscript.

Data sharing:

The data used for this study are subject to data sharing agreements with NHS Digital and Yorkshire Ambulance Service which prohibits further sharing of individual level data. The data sets used are obtainable from these organisations subject to necessary authorisations and approvals.

Figures:

Figure 1: STROBE flow diagram of selection of study population

For peer review only

For peer review only - http://bmjopen.bmj.com/site/about/guidelines.xhtml

References:

1. https://www.hsj.co.uk/emergency-care/thousands-of-londons-999-calls-answered-by-other-regions/7029302.article.
2. https://www.nationalleadership.gov.uk/supporting-the-nhs-in-its-hour-of-need/london-ambulance-service/.
3. Jensen T, Holgersen MG, Jespersen MS, et al. Strategies to Handle Increased Demand in the COVID-19 Crisis: A Coronavirus EMS Support Track and a Web-Based Self-Triage System. *Prehospital Emergency Care.* 2021;25:28-38.
4. Snooks H, Watkins AJ, Bell F, et al. Call volume, triage outcomes, and protocols during the first wave of the COVID-19 pandemic in the United Kingdom: Results of a national survey. *Journal of the American College of Emergency Physicians Open.* 2021;2:e12492.
5. Ferron R, Agarwal G, Cooper R, Munkley D. The effect of COVID-19 on emergency medical service call volumes and patient acuity: a cross-sectional study in Niagara, Ontario. *BMC Emergency Medicine.* 2021;21:39.
6. Charlton K, Limmer M, Moore H. Incidence of emergency calls and out-of-hospital cardiac arrest deaths during the COVID-19 pandemic: findings from a cross-sectional study in a UK ambulance service. *Emergency Medicine Journal.* 2021;38:446.
7. Blackwell TH, Kaufman JS. Response time effectiveness: comparison of response time and survival in an urban emergency medical services system. *Academic Emergency Medicine.* 2002;9:288-295.
8. https://www.london.gov.uk/press-releases/mayoral/hospitals-at-risk-of-being-overwhelmed-in-capital.
9. https://www.gov.uk/government/publications/covid-19-guidance-for-ambulance-trusts/covid-19-guidance-for-ambulance-trusts.
10. https://www.emergencydispatch.org/in-the-news/covid-19-communications/a332c5d1-0dbf-4354-9a64-8d149f75f15d.
11. Tankel JW, Ratcliffe D, Smith M, et al. Consequences of the emergency response to COVID-19: a whole health care system review in a single city in the United Kingdom. *BMC Emergency Medicine.* 2021;21:55.
12. https://digital.nhs.uk/services/national-data-opt-out.
13. Campbell MJ, Jacques RM, Fotheringham J, Maheswaran R, Nicholl J. Developing a summary hospital mortality index: retrospective analysis in English hospitals over five years. *BMJ.* 2012;344:e1001.
14. Street A, Maynou L, Gilbert T, Stone T, Mason S, Conroy S. The use of linked routine data to optimise calculation of the Hospital Frailty Risk Score on the basis of previous hospital admissions: a retrospective observational cohort study. *The Lancet Healthy Longevity.* 2021;2:e154-e162.
15. Rockwood K, Song X, MacKnight C, et al. A global clinical measure of fitness and frailty in elderly people. *CMAJ.* 2005;173:489-495.
16. Hirst E, Irving A, Goodacre S. Patient and public involvement in emergency care research. *Emergency Medicine Journal.* 2016;33:665-670.
17. https://digital.nhs.uk/binaries/content/assets/website-assets/publications/publications-admin-pages/related-documents/disclosure-control-procedure.pdf.
18. Marincowitz C, Stone T, Bath P, et al. Accuracy of telephone triage for predicting adverse outcome in suspected COVID-19: An observational cohort study. *medRxiv.* 2021:2021.2006.2024.21259441.
19. Goodacre S, Thomas B, Lee E, et al. Characterisation of 22445 patients attending UK emergency departments with suspected COVID-19 infection: Observational cohort study. *PLOS ONE.* 2020;15:e0240206.

For peer review only - http://bmjopen.bmj.com/site/about/guidelines.xhtml

20. https://digital.nhs.uk/data-and-information/publications/statistical/health-survey-for-england/2019.

21. Zachariasse JM, van der Hagen V, Seiger N, Mackway-Jones K, van Veen M, Moll HA. Performance of triage systems in emergency care: a systematic review and meta-analysis. *BMJ Open.* 2019;9:e026471.

22. Thomas B, Goodacre S, Lee E, et al. Prognostic accuracy of emergency department triage tools for adults with suspected COVID-19: the PRIEST observational cohort study. *Emergency Medicine Journal.* 2021:emermed-2020-210783.

23. https://www.yorkpress.co.uk/news/19012031.yorkshire-ambulance-service-declares-major-incident---report/.

24. https://www.reuters.com/article/us-health-coronavirus-britain-manchester-idUKKBN27I2HK.

25. https://cdn.emergencydispatch.org/iaed/pdf/NAE-Protocol-36-Flu-v13_3.pdf.

26. Goodacre S, Thomas B, Sutton L, et al. Derivation and validation of a clinical severity score for acutely ill adults with suspected COVID-19: The PRIEST observational cohort study. *PLoS One.* 2021;16:e0245840.

27. Abbasi J. Study Suggests Lasting Immunity After COVID-19, With a Big Boost From Vaccination. *JAMA.* 2021;326:376-377.

For peer review only

For peer review only - http://bmjopen.bmj.com/site/about/guidelines.xhtml

Figure 1: STROBE flow diagram of study population selection (Created by the authors)

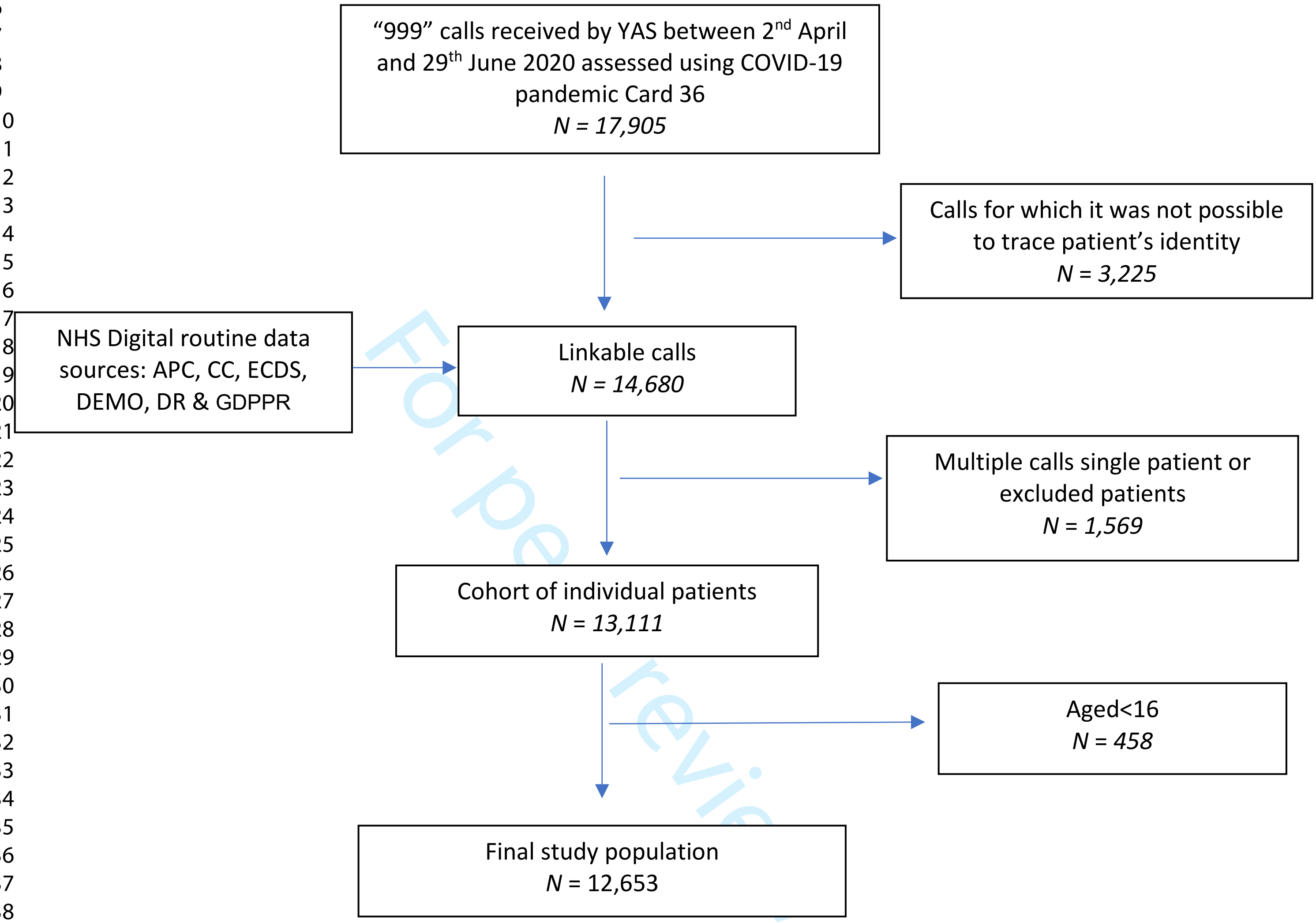

For peer review only

Supplementary Material 1: Summary of AMPDS pandemic Card 36 (Created by the authors) (full details available: https://cdn.emergencydispatch.org/iaed/pdf/resource-library/public%20protocols/pub-protocol%2036/NAE%20CC%2036.pdf)

Questions	Outcome
1) What is the most prominent complaint?	**Delta Category (immediate response)**<br>**Category 1/2 UK Ambulance service response**<br>1) INEFFECTIVE BREATHING with flu-like symptoms<br>2) DIFFICULTY SPEAKING BETWEEN BREATHS with flu-like symptoms<br>3) Not alert with flu-like symptoms<br>4) CHANGING COLOR with flu-like symptoms
If breathing a)Do they have difficulty speaking between breathes?<br>b) Describe their breathing<br>c)Did they have any flu symptoms prior to this?	
If Chest pain a) Are they 35 and over?<br>b) Have they had a heart attack or angina previously	
2) Are they completely alert?	
3) Are they changing colour?	
4) Are they having chills or sweats?	**Charlie Category (Ambulance when able)**<br>**Category 3/4 UK Ambulance service response**<br>1) Abnormal breathing with single flu-like symptom or Asthma/COPD<br>2) Abnormal breathing with multiple flu-like symptoms<br>3) Chest pain/discomfort ≥35 with single flu-like symptom<br>4) Chest pain/discomfort ≥35 with multiple flu-like symptoms<br>5) High Risk Factor
5) Are they vomiting?	
6)Do they have a new cough that recently started?	
7)Do they have a sore throat?	
8)Do they have unusual total body aches?	
9)Do they have a fever?	**Alpha Category (No ambulance dispatched)**<br>1) Chest pain/discomfort < 35 with single flu-like symptom<br>2) Chest pain/discomfort < 35 with multiple flu-like symptoms<br>3) Flu-like symptoms only
10)Do they have a runny or snotty nose?	
11)Do they have diarrhoea?	
12)Do they have a headache?	
13)Do they have any high-risk conditions?<br>• ≤ 5 years old (not COVID-19)<br>• ≥ 65 years old<br>• Blood disorders<br>• Diabetes<br>• Kidney and liver diseases/disorders<br>• Neurological diseases<br>• Pregnancy (up to 2 weeks after delivery)<br>• Sickle cell disease (sickle cell anaemia)<br>• Weakened immune system	

For peer review only - http://bmjopen.bmj.com/site/about/guidelines.xhtml

Supplementary Material 2: Multi-variable model predicting false negatives (Created by the authors)

Population Characteristic	Level	Odds ratio (95% CI) N= 1215
**Age (Years)**	1-year increase	0.95 (0.94 to 0.97)
**Sex**	Female	1.89 (1.09 to 3.26)
**Comorbidity**	Cardiovascular Disease	0.73 (0.21 to 2.56)
	Chronic Resp. Disease	0.50 (0.23 to 1.09)
	Diabetes	1.19 (0.60 to 2.38)
	Hypertension	0.75 (0.39 to 1.46)
	Immunosuppression (including steroid use)	0.73 (0.35 to 1.53)
	Active Malignancy	0.12 (0.02 to 0.92)
	Obesity	Not included
	Renal Impairment	2.22 (0.71 to 6.96)
	Smoker	0.96 (0.52 to 1.80)
	Stroke	1.93 (0.42 to 8.80)
**Number of Drugs Used**	0	Reference
	1-5	0.98 (0.45 to 2.15)
	6-10	1.56 (0.55 to 4.44)
	11 or more	0.76 (0.08 to 7.42)
**Deprivation Index**	1-2	Reference
	3-4	0.98 (0.47 to 2.08)
	5-6	0.54 (0.21 to 1.36)
	7-8	1.37 (0.64 to 2.97)
	9-10	0.99 (0.38 to 2.59)

For peer review only

Supplementary Material 3: Multi-variable model predicting false positives

Population Characteristic	Level	Odds ratio (95% CI) N= 5, 615
**Age (Years)**	1-year increase	1.05 (1.04 to 1.05)
**Sex**	Female	1.31 (1.15 to 1.49)
**Comorbidity**	Cardiovascular Disease	1.38 (0.85 to 2.24)
	Chronic Resp. Disease	1.35 (1.13 to 1.60)
	Diabetes	0.85 (0.67 to 1.09)
	Hypertension	1.03 (0.84 to 1.26)
	Immunosuppression (including steroid use)	1.02 (0.82 to 1.26)
	Active Malignancy	1.25 (0.72 to 2.18)
	Renal Impairment	0.80 (0.44 to 1.44)
	Smoker	0.86 (0.75 to 0.99)
	Stroke	1.17 (0.59 to 2.31)
**Number of Drugs Used**	0	Reference
	1-5	0.99 (0.84 to 1.16)
	6-10	1.22 (0.90 to 1.66)
	11 or more	1.13 (0.62 to 2.06)
**Deprivation Index**	1-2	Reference
	3-4	1.21 (1.02 to 1.44)
	5-6	1.23 (1.01 to 1.48)
	7-8	1.29 (1.05 to 1.59)
	9-10	1.32 (1.04 to 1.69)

For peer review only

The RECORD statement – checklist of items, extended from the STROBE statement, that should be reported in observational studies using routinely collected health data.

	Item No.	STROBE items	Location in manuscript where items are reported	RECORD items	Location in manuscript where items are reported
**Title and abstract**					
	1	(a) Indicate the study's design with a commonly used term in the title or the abstract (b) Provide in the abstract an informative and balanced summary of what was done and what was found	Pages 1-2	RECORD 1.1: The type of data used should be specified in the title or abstract. When possible, the name of the databases used should be included.<br><br>RECORD 1.2: If applicable, the geographic region and timeframe within which the study took place should be reported in the title or abstract.<br><br>RECORD 1.3: If linkage between databases was conducted for the study, this should be clearly stated in the title or abstract.	Page 2 (abstract)
**Introduction**					
Background rationale	2	Explain the scientific background and rationale for the investigation being reported	Page 3		
Objectives	3	State specific objectives, including any prespecified hypotheses	Page 3		
**Methods**					
Study Design	4	Present key elements of study design early in the paper	Page 4		
Setting	5	Describe the setting, locations, and relevant dates, including periods of recruitment, exposure, follow-up, and data collection	Page 4		

For peer review only

Participants	6	*(a) Cohort study* - Give the eligibility criteria, and the sources and methods of selection of participants. Describe methods of follow-up<br>*Case-control study* - Give the eligibility criteria, and the sources and methods of case ascertainment and control selection. Give the rationale for the choice of cases and controls<br>*Cross-sectional study* - Give the eligibility criteria, and the sources and methods of selection of participants<br><br>*(b) Cohort study* - For matched studies, give matching criteria and number of exposed and unexposed<br>*Case-control study* - For matched studies, give matching criteria and the number of controls per case	Page 4,5	RECORD 6.1: The methods of study population selection (such as codes or algorithms used to identify subjects) should be listed in detail. If this is not possible, an explanation should be provided.<br><br>RECORD 6.2: Any validation studies of the codes or algorithms used to select the population should be referenced. If validation was conducted for this study and not published elsewhere, detailed methods and results should be provided.<br><br>RECORD 6.3: If the study involved linkage of databases, consider use of a flow diagram or other graphical display to demonstrate the data linkage process, including the number of individuals with linked data at each stage.	Page 4,5<br>Figure 1
Variables	7	Clearly define all outcomes, exposures, predictors, potential confounders, and effect modifiers. Give diagnostic criteria, if applicable.	Page 5,6	RECORD 7.1: A complete list of codes and algorithms used to classify exposures, outcomes, confounders, and effect modifiers should be provided. If these cannot be reported, an explanation should be provided.	
Data sources/ measurement	8	For each variable of interest, give sources of data and details of methods of assessment (measurement).<br>Describe comparability of assessment methods if there is more than one group	Page 5,6		

For peer review only

Bias	9	Describe any efforts to address potential sources of bias	Page 5-6		
Study size	10	Explain how the study size was arrived at	Pages 4,5 Figure 1		
Quantitative variables	11	Explain how quantitative variables were handled in the analyses. If applicable, describe which groupings were chosen, and why	Pages 5,6		
Statistical methods	12	(a) Describe all statistical methods, including those used to control for confounding<br>(b) Describe any methods used to examine subgroups and interactions<br>(c) Explain how missing data were addressed<br>(d) *Cohort study* - If applicable, explain how loss to follow-up was addressed<br>*Case-control study* - If applicable, explain how matching of cases and controls was addressed<br>*Cross-sectional study* - If applicable, describe analytical methods taking account of sampling strategy<br>(e) Describe any sensitivity analyses	Pages 5,6		
Data access and cleaning methods		..	Page 4	RECORD 12.1: Authors should describe the extent to which the investigators had access to the database population used to create the study population.	Page 4

For peer review only

				RECORD 12.2: Authors should provide information on the data cleaning methods used in the study.	
Linkage		..	Page 4	RECORD 12.3: State whether the study included person-level, institutional-level, or other data linkage across two or more databases. The methods of linkage and methods of linkage quality evaluation should be provided.	Figure 1
**Results**					
Participants	13	(a) Report the numbers of individuals at each stage of the study (*e.g.*, numbers potentially eligible, examined for eligibility, confirmed eligible, included in the study, completing follow-up, and analysed)<br>(b) Give reasons for non-participation at each stage.<br>(c) Consider use of a flow diagram	Figure 1	RECORD 13.1: Describe in detail the selection of the persons included in the study (*i.e.,* study population selection) including filtering based on data quality, data availability and linkage. The selection of included persons can be described in the text and/or by means of the study flow diagram.	Figure 1
Descriptive data	14	(a) Give characteristics of study participants (*e.g.*, demographic, clinical, social) and information on exposures and potential confounders<br>(b) Indicate the number of participants with missing data for each variable of interest<br>(c) *Cohort study* - summarise follow-up time (*e.g.*, average and total amount)	Table 1		
Outcome data	15	*Cohort study* - Report numbers of outcome events or summary measures over time<br>*Case-control study* - Report numbers in each exposure	Page 6-8		

For peer review only

		category, or summary measures of exposure *Cross-sectional study* - Report numbers of outcome events or summary measures			
Main results	16	(a) Give unadjusted estimates and, if applicable, confounder-adjusted estimates and their precision (e.g., 95% confidence interval). Make clear which confounders were adjusted for and why they were included (b) Report category boundaries when continuous variables were categorized (c) If relevant, consider translating estimates of relative risk into absolute risk for a meaningful time period	Pages 8-11		
Other analyses	17	Report other analyses done—e.g., analyses of subgroups and interactions, and sensitivity analyses	Pages 8-11		
**Discussion**					
Key results	18	Summarise key results with reference to study objectives	Pages 12		
Limitations	19	Discuss limitations of the study, taking into account sources of potential bias or imprecision. Discuss both direction and magnitude of any potential bias	Pages 12-13	RECORD 19.1: Discuss the implications of using data that were not created or collected to answer the specific research question(s). Include discussion of misclassification bias, unmeasured confounding, missing data, and changing eligibility over time, as they pertain to the study being reported.	Pages 12-13
Interpretation	20	Give a cautious overall interpretation of results considering objectives,	Pages 13-14		

		limitations, multiplicity of analyses, results from similar studies, and other relevant evidence			
Generalisability	21	Discuss the generalisability (external validity) of the study results	Pages 12-14		
**Other Information**					
Funding	22	Give the source of funding and the role of the funders for the present study and, if applicable, for the original study on which the present article is based	15		
Accessibility of protocol, raw data, and programming code		..	15	RECORD 22.1: Authors should provide information on how to access any supplemental information such as the study protocol, raw data, or programming code.	15

*Reference: Benchimol EI, Smeeth L, Guttmann A, Harron K, Moher D, Petersen I, Sørensen HT, von Elm E, Langan SM, the RECORD Working Committee. The REporting of studies Conducted using Observational Routinely-collected health Data (RECORD) Statement. *PLoS Medicine* 2015; in press.

*Checklist is protected under Creative Commons Attribution (CC BY) license.

For peer review only

BMJ Open

Accuracy of emergency medical service telephone triage of need for an ambulance response in suspected COVID-19: An observational cohort study

Journal:	*BMJ Open*
Manuscript ID	bmjopen-2021-058628.R1
Article Type:	Original research
Date Submitted by the Author:	25-Mar-2022
Complete List of Authors:	Marincowitz, Carl; The University of Sheffield, Centre for Urgent and Emergency Care Research<br>Stone, Tony; The University of Sheffield, Centre for Urgent and Emergency Care Research<br>Hasan, Madina ; The University of Sheffield, Centre for Urgent and Emergency Care Research<br>Campbell, Richard; The University of Sheffield, Centre for Urgent and Emergency Care Research<br>Bath, Peter; The University of Sheffield, Information School<br>Turner, Janette; The University of Sheffield, Centre for Urgent and Emergency Care Research<br>Pilbery, Richard; Yorkshire Ambulance Service NHS Trust, Research and Development<br>Thomas, Benjamin; The University of Sheffield, Clinical Trials Research Unit<br>Sutton, Laura ; The University of Sheffield, Clinical Trials Research Unit<br>Bell, Fiona; Yorkshire Ambulance Service NHS Trust, Research and Development<br>Biggs, Katie; The University of Sheffield, Clinical Trials Research Unit<br>Hopfgartner, Frank; The University of Sheffield, Information School<br>Mazumdar, Suvodeep; The University of Sheffield, Information School<br>Petrie, Jennifer; The University of Sheffield, Clinical Trials Research Unit<br>Goodacre, Steve; The University of Sheffield, Centre for Urgent and Emergency Care Research
**Primary Subject Heading**:	Emergency medicine
Secondary Subject Heading:	Respiratory medicine, Health services research
Keywords:	COVID-19, Risk management < HEALTH SERVICES ADMINISTRATION & MANAGEMENT, ACCIDENT & EMERGENCY MEDICINE

SCHOLARONE™
Manuscripts

I, the Submitting Author has the right to grant and does grant on behalf of all authors of the Work (as defined in the below author licence), an exclusive licence and/or a non-exclusive licence for contributions from authors who are: i) UK Crown employees; ii) where BMJ has agreed a CC-BY licence shall apply, and/or iii) in accordance with the terms applicable for US Federal Government officers or employees acting as part of their official duties; on a worldwide, perpetual, irrevocable, royalty-free basis to BMJ Publishing Group Ltd ("BMJ") its licensees and where the relevant Journal is co-owned by BMJ to the co-owners of the Journal, to publish the Work in this journal and any other BMJ products and to exploit all rights, as set out in our licence.

The Submitting Author accepts and understands that any supply made under these terms is made by BMJ to the Submitting Author unless you are acting as an employee on behalf of your employer or a postgraduate student of an affiliated institution which is paying any applicable article publishing charge ("APC") for Open Access articles. Where the Submitting Author wishes to make the Work available on an Open Access basis (and intends to pay the relevant APC), the terms of reuse of such Open Access shall be governed by a Creative Commons licence – details of these licences and which Creative Commons licence will apply to this Work are set out in our licence referred to above.

Other than as permitted in any relevant BMJ Author's Self Archiving Policies, I confirm this Work has not been accepted for publication elsewhere, is not being considered for publication elsewhere and does not duplicate material already published. I confirm all authors consent to publication of this Work and authorise the granting of this licence.

For peer review only

Accuracy of emergency medical service telephone triage of need for an ambulance response in suspected COVID-19: An observational cohort study

Carl Marincowitz[1] NIHR Clinical Lecturer Emergency Medicine

Tony Stone[1] Senior Data Manager

Madina Hasan[1] Research Associate

Richard Campbell[1] Data Specialist

Peter A. Bath[1,2] Professor of Health Informatics and Health Data Analytics

Janette Turner[1] Honorary Senior Research Fellow

Richard Pilbery[3] Research paramedic

Benjamin Thomas[4] Study Manager

Laura Sutton[4] Research Associate

Fiona Bell[3] Head of Research

Katie Biggs[4] Research Fellow/Assistant CTRU Director

Frank Hopfgartner[2] Senior Lecturer in Data Science

Suvodeep Mazumdar[2] Lecturer in Data Analytics

Jennifer Petrie[4] Trial Manager

Steve Goodacre[1] Professor of Emergency Medicine

1. Centre for Urgent and Emergency Care Research (CURE), Health Services Research School of Health and Related Research, University of Sheffield, Regent Court, 30 Regent Street, Sheffield, S1 4DA, UK, Fax: +44 (0)114 222 0749 Tel: (+44) (0)114 222 4345, Email: C.Marincowitz@Sheffield.ac.uk
2. Information School, University of Sheffield, Regent Court, 211 Portobello St, Sheffield S1 4DP, UK
3. Yorkshire Ambulance Service NHS Trust, Trust HQ, Springhill 2, Brindley Way, Wakefield, United Kingdom, WF2 0XQ
4. Clinical Trials Research Unit (CTRU), Health Services Research School of Health and Related Research, University of Sheffield, Regent Court, 30 Regent Street, Sheffield, S1 4DA, UK, Fax: +44 (0)114 222 0749 Tel: (+44) (0)114 222 4345,

Correspondence to Dr Benjamin Thomas at b.d.thomas@sheffield.ac.uk

For peer review only - http://bmjopen.bmj.com/site/about/guidelines.xhtml

Abstract

Objective: To assess accuracy of Emergency Medical Service (EMS) telephone triage in identifying patients who need an EMS response and identify factors which affect triage accuracy.

Design: Observational cohort study

Setting: Emergency telephone triage provided by Yorkshire Ambulance Service NHS Trust (YAS).

Participants: 12, 653 adults who contacted EMS telephone triage services provided by YAS between the 2nd April 2020 and 29th June 2020 assessed by COVID-19 telephone triage pathways were included.

Outcome: Accuracy of call handler decision to dispatch an ambulance was assessed in terms of death or need for organ support at 30 days from first contact with the telephone triage service.

Results: Callers contacting EMS dispatch services had an 11.1% (1,405/12,653) risk of death or needing organ support. In total, 1,335/12,653 (16%) of callers did not receive an emergency response and they had a 70/1,335 (3.5%) risk of death or organ support. Ambulances were dispatched to 4, 230 callers (33.4%) who were not conveyed to hospital and did not deteriorate. Multivariable modelling found variables of older age (1 year increase OR 1.05, 95% CI:1.04 to 1.05) and presence of pre-existing respiratory disease (OR 1.35 95% CI:1.13 to 1.60) to be predictors of false positive triage.

Conclusion: Telephone triage can reduce ambulance responses but, with low specificity. A small but significant proportion of patients who do not receive an initial emergency response deteriorated. Research to improve accuracy of EMS telephone triage is needed and, due to limitations of routinely collected data, this is likely to require prospective data collection.

Strengths and limitations of this study

- Despite concerns regarding accuracy in identifying need for emergency treatment, this is one of the first evaluations of EMS telephone triage protocols for suspected COVID-19.
- Use of ambulance data linked to nationally collected death registrations and routinely collected healthcare data provided robust outcome data for all included callers.
- Use of routine data limited assessment of factors which affect triage accuracy to those which are routinely collected.
- The evaluation in this study is of a single United Kingdom (UK) ambulance service's implementation of EMS telephone triage protocols for suspected COVID-19 cases.

Word Count: 3, 248

For peer review only - http://bmjopen.bmj.com/site/about/guidelines.xhtml

Background

During the COVID-19 pandemic, emergency medical service (EMS) call volumes have been volatile in the United Kingdom (UK), Europe and North America. Some ambulance services in the UK reported up to three times the expected number of EMS calls during the first and second waves of the pandemic, an increase also observed in other parts of Europe.[1-4] Other EMS providers in the UK and North America observed initial decreases in call volumes.[5, 6] Surges in demand in the second wave of the pandemic led to some ambulance services in the UK declaring major incidents and warning of care being compromised by overwhelming demand.[7, 8]

EMS call handlers in the UK are typically trained non-clinical staff who use either the Advanced Medical Priority Dispatch System (AMPDS) or NHS Pathways to triage the EMS response to calls. [4] On the 2nd April 2020, six English NHS ambulance services using AMPDS introduced a specific protocol for callers with suspected COVID-19 using the pandemic Card 36.[9, 10] Structured questions (summarised in Supplementary Material 1) were used to triage urgency of EMS response into three levels: Delta (highest priority requiring immediate response), Charlie (medium priority requiring ambulance attendance when able) and Alpha (no ambulance dispatched). The AMPDS Card 36 was implemented by UK ambulance services in different ways, with some symptoms and selected high risk-groups receiving a higher priority response than normal, with alterations to the card implemented in the following months.[4]

There is evidence that changes in EMS practice due to the pandemic and delays in the emergency assessment of patients with COVID-19 may have contributed to avoidable deaths in the North West of England.[11] Despite the need for EMS telephone triage to balance ambulance responses to patients requiring life-saving interventions against an anticipation of overwhelming demand during the pandemic, there has been no previous evaluation of the accuracy of the clinical risk-assessment performed for patients with suspected COVID-19.

Our study aimed to:

1) Assess how accurately EMS telephone triage identified those likely to suffer an adverse outcome (death or organ support) and subsequently require an emergency ambulance response.
2) Identify any factors that may have affected the accuracy of EMS telephone triage.

Methods

Study Design and Setting

For peer review only - http://bmjopen.bmj.com/site/about/guidelines.xhtml

This observational cohort study used linked routinely collected EMS telephone call centre data from Yorkshire Ambulance Service NHS Trust (YAS) to assess the accuracy of clinical triage of patients with suspected COVID-19.

Emergency services provided by YAS covers a region in the north of England of approximately 6,000 square miles and with a population of 5.3 million. In 2020/2021, YAS received more than 1,000,000 emergency (999) calls.

Data Sources and linkage

Yorkshire Ambulance Service provided a dataset of all 999 calls, triaged using a Card 36 pandemic triage pathway for patients with suspected COVID-19, received between the 2ndApril and 29th June 2020. The dataset consisted of patient identifiers, demographic data, call details and the outcome of the call (including whether an ambulance was dispatched) extracted from routinely collected electronic call records.

Health and social care data relating to the population in England within the UK National Health Service (NHS) are managed by NHS Digital. We provided patient identifiers to NHS Digital to trace patients in our cohort and supply additional individual-level demographic, co-morbidity and outcome data. NHS Digital identified records in their collections belonging to patients in our cohort, and provided data on patient demographics, limited COVID-related general practice (GP) records, emergency department attendances, hospital inpatient admissions, critical care periods, and death registrations from the UK Office for National Statistics (ONS).

YAS and NHS Digital removed records where patients indicated that they did not wish their data to be used for research purposes, via the NHS data opt-out service.[12] The study team also excluded patients who had opted out of any part of the wider PRIEST study,[13] of which this evaluation forms a part, and those with inconsistent records (e.g., multiple deaths recorded or death before latest activity). Patient identifiers across all datasets were replaced with a consistent pseudo-identifier to enable the identification and linkage of records belonging to the same patient across all datasets but without revealing any patient identifiers. Calls which originated from other health care services (i.e., where a decision that ambulance dispatch was required had already been made) were excluded from the study population.

Inclusion criteria

Our final cohort consisted of all adult (aged 16 years and over) callers at time of first (index) EMS 999 call between 2nd April and 29th June 2020 assessed using the suspected COVID-19 Card 36 triage pathway, who were successfully traced by NHS Digital.

For peer review only - http://bmjopen.bmj.com/site/about/guidelines.xhtml

Outcome

The primary outcome was death, renal, respiratory, or cardiovascular organ support (identified from death registration and critical care data) at 30 days from index contact.

The secondary outcomes were hospital conveyance following ambulance dispatch (transfer to ED or inpatient setting) and inpatient admission (recoded inpatient dataset) 30 days from index contact. The 30-day duration of outcomes was based on the duration used when deriving the PRIEST clinical severity score.[13]

Patient Characteristics

Consistent with methods used to estimate the Charlson comorbidity index from the available routine data, comorbidities were included if recorded within 12 months before the index EMS call.[14, 15] In a similar way, only immunosuppressant drug prescriptions documented in GP records within 30 days before the index contact, contributed to the immunosuppression co-morbidity variable. Pregnancy status was based on GP records recorded in the previous 9 months. Frailty in patients older than 65 years was derived from the latest recorded Clinical Frailty Scale (CFS) score (if recorded) in the electronic GP records prior to index attendance.[16] Patients under the age of 65 years were not given a CFS score since it is not validated in this age group. However, in multivariable analysis, patients under the age of 65 were assumed to have a functional level equivalent to a mild frailty category (CFS 1-3).

Analysis

We conducted a descriptive analysis of caller demographics, co-morbidities and call disposition. The proportion of callers who experienced the primary and secondary outcome was estimated. To assess the accuracy of EMS telephone triage in identifying clinical outcomes requiring an emergency response, the call disposition of the index contact was divided into the binary classification of either: ambulance dispatched (Delta or Charlie priority); or other call outcome (Alpha priority) (Supplementary Material 1). We assessed the accuracy of the binary triage classification (ambulance dispatched versus no ambulance dispatched) in terms of sensitivity, specificity, positive predictive value (PPV) and negative predictive value (NPV) for the primary and secondary outcomes with 95% confidence intervals (CI).

Patient characteristics of false negatives (those who experienced the primary outcome (death or organ support) and no ambulance was dispatched) and true positives (ambulance dispatched who

For peer review only - http://bmjopen.bmj.com/site/about/guidelines.xhtml

experienced the primary outcome) were compared. In patients with the adverse outcome (death or organ support) multi-variable logistic regression was used to identify patient characteristics associated with false negative triage. We also compared the characteristics of false positives (ambulance dispatched and not conveyed to hospital) and true negatives (no ambulance dispatched) among those who did not experience the primary composite outcome. We used multivariable logistic regression to identify factors which predicted false positive EMS triage. Obesity was excluded from multivariable analysis due to an observed implausible protective association with the primary outcome (death or organ support), which we believe to be an artefact of how these data were collected and recorded in the electronic GP data set.[17, 18] Ethnicity and frailty were also excluded from multivariable analysis due to the high proportion of missing data. All analyses were completed using STATA 16 (StataCorp. 2019. *Stata Statistical Software: Release 16*. College Station, TX: StataCorp LLC).

Ethical Approval

The North West—Haydock Research Ethics Committee gave a favourable opinion on the PAINTED study on 25th June 2012 (reference 12/NW/0303) and on the updated PRIEST study on 23rd March 2020, including the analyses presented here. The Confidentiality Advisory Group of the NHS Health Research Authority granted approval to collect data without patient consent in line with Section 251 of the National Health Service Act 2006. Access to data collected by NHS Digital was recommended for approval by its Independent Group Advising on the Release of Data (IGARD) on 11th September 2021, having received additional recommendation for approval for access to GP records from the Profession Advisory Group (PAG) on 19th August 2021.

Patient Public Involvement

The Sheffield Emergency Care Forum (SECF) is a public representative group interested in emergency care research.[19] Members of SECF advised on the development of the PRIEST study and two members joined the Study Steering Committee. Patients were not involved in the conduct of the study.

Results

All totals (including outcome) presented are rounded to the nearest 5, with small numbers suppressed to comply with NHS Digital data disclosure guidance.[20]

For peer review only - http://bmjopen.bmj.com/site/about/guidelines.xhtml

Study population

Figure 1 and Table 1 summarise study cohort derivation and the characteristics of the 12, 653 included individual callers. In total, 1,405 callers (11.1%, 95% CI: 10.5 to 11.7%) experienced the primary outcome (death or organ support) within 30 days following the index EMS 999 call. An ambulance was dispatched to 10,650 (84.2%) of callers. In our study cohort, 6,070 patients (48%. 95%: 47.1 to 48.9%) were conveyed to hospital (ED or directly to an inpatient setting) and 5,735 (45.3%, 95% CI: 44.5 to 46.2%) were admitted as hospital inpatients within 30 days of index contact.

The median age of the whole cohort was 66 (IQR = 46-81), the cohort had an almost equal proportion of males (49.5%) and females (50.5%) and had high rates of comorbidity (chronic respiratory disease 29.5%, diabetes 16.6% and hypertension 35.9%).

Table 1: Population characteristics

Population Characteristic	Level	Whole Population N=12, 653	No Death or organ support (30 days) N= 11,250	Death or organ support(30 days) N=1,405
**Age (Years)**	Median (IQR)*<br>Mean	66 (46-81)<br>62.3	63 (43-79)<br>60.6	80 (67-87)<br>70
**Sex (N, %)**	Male	6,260 (49.5%)	5,465 (48.6%)	795 (56.5%)
**Comorbidity (N, %)**	Cardiovascular Disease	810 (6.4%)	690 (6.1%)	120 (8.6%)
	Chronic Resp. Disease	3,730 (29.5%)	3,320 (29.5%)	410 (29.4%)
	Diabetes	2,095 (16.5%)	1,770 (10.2%)	325 (23.1%)
	Hypertension	4,545 (35.9%)	6,760 (15.7%)	705 (50.4%)
	Immunosuppression (including steroid use)	2, 540 (20.1%)	3,545 (31.5%)	360 (25.8%)
	Active Malignancy	595 (4.7%)	435 (3.9%)	160 (11.5%)
	Obesity	1,960 (15.5%)	1,860 (16.5%)	100 (7.3%)
	Pregnant	135 (1.1%)	135 (1.2%)	-
	Renal Impairment	365 (2.9%)	310 (2.7%)	60 (4.3%)
	Stroke	285 (2.2%)	240 (2.1%)	45 (3.1%)
**Social (N, %)**	Smoker	4,810 (38.0%)	1,740 (15.5%)	550 (39.3%)
**Number of Prescribed Drugs Used**	0	3,115 (24.6%)	2,940 (15.1)	175 (12.4%)
	1-5	6,015 (47.5%)	5,320 (47.3%)	700 (49.8%)
	6-10	3,030 (23.9%)	2,565 (22.8%)	465 (33.1%)

For peer review only - http://bmjopen.bmj.com/site/about/guidelines.xhtml

**(N, %)**	11 or more	490 (3.9%)	425 (3.8%)	65 (4.7%)
**Clinical Frailty Scale (N, %)**	Unknown	4,560 (36.0%)	3,875 (34.4%)	685 (48.9%)
	Aged<65	6,105 (48.2%)	5,800 (51.5%)	305 (21.6%)
	1-3	165 (1.3%)	150 (1.3%)	15 (1.0%)
	3-6	670 (5.3%)	590 (5.2%)	85 (6.0%)
	6-9	1,155 (9.1%)	840 (7.5%)	315 (22.5%)
**Ethnicity (N, %)**	Asian or Asian British	750 (5.9%)	700 (6.2%)	5 (3.6%)
	Black or Black British	190 (1.5%)	180 (1.6%)	15 (0.9%)
	Mixed	110 (0.9%)	105 (0.9%)	-
	Other Ethnic Groups	160 (1.3%)	150 (1.3%)	-
	White	9,020 (71.3%)	8,070 (71.7%)	950 (67.8%)
	Unknown	2,425 (19.2%)	2,050 (18.2%)	375 (26.6%)
**Deprivation Index (N, %)**	Unknown	1,250 (9.9%)	1,060 (9.4%)	190 (13.5%)
	1-2	4,600 (40.3%)	4,155 (40.8%)	440 (36.4%)
	3-4	2,075 (18.2%)	1,855 (18.2%)	220 (18%)
	5-6	1,880 (16.5%)	1,660 (16.3%)	220 (18%)
	7-8	1,695 (14.9%)	1,485 (14.6%)	210 (17.2%)
	9-10	1,160 (10.2%)	1,030 (10.1%)	130 (10.6%)
**Ambulance Dispatched (N, %)**	Ambulance	10,650 (84.2%)	9,320 (82.8%)	1,335 (95%)
	No Ambulance	2,000 (15.8%)	1,930 (17.2%)	70 (5%)
**Outcome (N, %)**	Death	1,155 (9.1%)	NA***	1,155 (82.4%)
	Deaths due to COVID (including after 30 days)	690 (5.5%)	NA	530 (37.8%)
	Organ support (within 30 days)	335 (2.6%)	NA	335 (23.8%)
**Hospitalisation (N, %)**	ED Attendance	6,945 (54.9%)	5,975 (53.1%)	970 (69.1%)
	Inpatient admission	5,735 (45.3%)	4,650 (41.3%)	1,085 (77.3%)
**Confirmed Hospital Diagnosis of COVID (N, %)****	In ED or as inpatient at 30 days	1,895 (15%)	1,300 (11.6%)	595 (42.3%)
**Time to Primary Outcome from index contact- up to and including (N, %)**	72 hours	475 (3.8%)	NA	475 (33.6%)
	7 days	780 (6.1%)	**NA**	780 (55.5%)

*Interquartile Range (IQR)

**Unrestricted community testing for suspected COVID-19 was only available from the 18/05/2020. Confirmed diagnosis is based upon inpatient PCR testing or clinical diagnosis in hospital

*** Not Applicable (NA)

- Suppressed due to small numbers

For peer review only - http://bmjopen.bmj.com/site/about/guidelines.xhtml

Accuracy of telephone triage of ambulance dispatch

Table 2 shows the accuracy of the EMS telephone triage decision to dispatch an ambulance for the composite primary outcome (death or organ support) indicating need for emergency intervention. Decision to dispatch an ambulance achieved a sensitivity of 95% (95% CI:93.7 to 96.1%) to the primary outcome. If advised to self-care/non-urgent clinical assessment, the chance of experiencing an adverse outcome (death or organ support) was approximately 3.5% (NPV: 96.5%, 95% CI: 95.6 to 97.2%). The high sensitivity was achieved at the cost of specificity (17.2% 95% CI: 16.5% to 17.9%). Among patients for whom an ambulance was dispatched, the risk of serious adverse outcomes was 12.5% (95% CI: 11.9 to 13.2%), transfer to hospital was 57% (95% CI: 56 to 58%) and hospital admission 49.3% (48.3 to 50.2%).

Table 2: Performance of decision to dispatch ambulance for composite primary outcome (death or organ support)

Primary outcome 30 days (11.1%, 10.5-11.7%)			
N= 12,653	**Death or organ support**	**No death or organ support**	
Ambulance dispatched	1,335	9,320	Sensitivity 95% (93.7-96.1%)<br>Positive Predictive Value 12.5% (11.9 – 13.2%)
No Ambulance dispatched	70	1,930	Specificity 17.2% (16.5% - 17.9%)<br>Negative Predictive Value 96.5% (95.6 - 97.2%)

Prediction of false negative or false positive ambulance dispatch

Table 3 compares the characteristics of patients who experienced the primary outcome (death or organ support), and either did (true positives) or did not (false negatives) have an ambulance dispatched on index call. In both groups, over 50% of people experienced the primary adverse outcome within seven days of first contact and around 33% of patients experienced the adverse outcome within 72 hours of index assessment. Multivariable modelling (Supplementary Material 2) showed that female sex (OR 1.89, 95% CI 1.09 to 3.26) was associated with increased risk and, increasing age (OR 0.95, 95% CI 0.94 to 0.97) and malignancy (OR 0.12, 95% CI 0.02 to 0.92) reduced risk, of false negative triage.

For peer review only - http://bmjopen.bmj.com/site/about/guidelines.xhtml

Table 3: False negatives compared to true positives

Population Characteristic	Level	False Negatives (No ambulance dispatch and primary outcome 30 days) N= 70	True Positives (Ambulance dispatch and primary outcome 30 days) N= 1,335
Age (Years)	Median (IQR)<br>Mean	61.5 (51-80)<br>64	80 (68-87)<br>76.7
Sex (N, %)	Male	35 (51.4%)	760 (56.8%)
Comorbidity (N, %)	Cardiovascular Disease	*	115 (8.8%)
	Chronic Resp. Disease	15 (20%)	400 (29.9%)
	Diabetes	20 (25.7%)	305 (23%)
	Hypertension	25 (34.3%)	685 (51.2%)
	Immunosuppression (including steroid use)	10 (17.1%)	350 (26.3%)
	Active Malignancy	*	160 (11.9%)
	Obesity	10 (12.9%)	95 (7%)
	Pregnant	*	*
	Renal Impairment	*	55 (4.2%)
	Stroke	*	40 (3.2%)
Social (N, %)	Smoker	25 (32.9%)	530 (39.6%)
Number of Drugs Used (N, %)	0	20 (25.7%)	155 (11.7%)
	1-5	30 (42.9%)	670 (50.2%)
	6-10	20 (28.6%)	445 (33.3%)
	11 or more	*	65 (4.8%)
Clinical Frailty Scale (N, %)	Unknown	60 (84.3%)	665 (49.8%)
	Aged<65	*	260 (19.7%)
	1-3	*	15 (1%)
	3-6	*	80 (6.2%)
	6-9	*	310 (23.4%)
Ethnicity (N, %)	Asian or Asian British	*	45 (3.5%)
	Black or Black British	*	15 (1%)
	Mixed	*	*
	Other Ethnic Groups	*	10 (0.7%)
	White	45 (61.4%)	910 (68.1%)
	Unknown	20 (28.6%)	355 (26.5%)
Deprivation Index (N, %)	Unknown	*	185 (13.7%)
	1-2	30 (46%)	410 (35.8%)
	3-4	10 (17.4%)	205 (17.9%)
	5-6	*	210 (18.4%)
	7-8	10 (17.4%)	200 (17.2%)
	9-10	*	120 (10.6%)
Outcome (N, %)	Death	45 (65.7%)	1,110 (83.3%)
	Deaths due to COVID (including after 30 days)	20 (27.2%)	520 (39%)
	Organ support (within 30 days)	35 (47.1%)	300 (22.6%)
Hospitalisation (N, %)	ED attendance	45	925

For peer review only - http://bmjopen.bmj.com/site/about/guidelines.xhtml

		(65.7%)	(69.2%)
	Inpatient admission	50 (72.9%)	1,035 (77.6%)
**Confirmed Hospital Diagnosis of COVID (N, %)****	In ED or as inpatient at 30 days	25 (34.3%)	570 (42.7%)
**Time to Primary Outcome from index contact- up to and including (N, %)**	72 hours	25 (37.1%)	445 (33.5%)
	7 days	45 (62.8%)	730 (54.9%)

Table 4 compares the characteristics of patients without the primary outcome (death or organ support) for whom an ambulance was dispatched and the patient was not transferred to hospital (False Positives), or no ambulance was dispatched (True Negatives). 33.4% of the cohort were false positives and Supplementary Material 3 presents the results of multivariable modelling to identify factors associated with false postivie triage. Risk of false positive was strongly associated with chronic respiratory disease (OR 1.35 95% CI 1.13 to 1.60) and reduced in smokers (OR 0.86, 95% CI 0.75 to 0.99). Increasing age, deprivation and female sex were also associated with risk of false positive triage.

Table 4: False positive compared to true negatives

Population Characteristic	Level	False Positive (Ambulance dispatched, not conveyed to hospital and no primary outcome 30 days) N= 4,230	True Negative (No ambulance dispatched and no primary outcome 30 days) N= 1,930
**Age (Years)**	Median (IQR)<br>Mean	66 (46-81)<br>62.9	37 (28-53)<br>41.6
**Sex (N, %)**	Male	1,920 (45.4%)	1000 (51.7%)
**Comorbidity (N, %)**	Cardiovascular Disease	235 (5.5%)	30 (1.6%)
	Chronic Resp. Disease	1,280 (30.3%)	380 (19.5%)
	Diabetes	600 (14.2%)	155 (7.8%
	Hypertension	1,480 (35%)	255(13.1%)
	Immunosuppression (including steroid use)	825 (19.5%)	180 (9.4%)
	Active Malignancy	140 (3.3%)	20 (0.9%)
	Obesity	700 (16.5%)	260 (13.4%)
	Pregnant	50 (1.2%)	40 (2.2%)
	Renal Impairment	110 (2.6%)	15 (0.8%)
	Stroke	90 (2.1%)	15 (0.7%)
**Smoking Status (N, %)**	Smoker	1,570 (37.1%)	640 (33.2%)
**Number of Drugs Used (N, %)**	0	1,040 (24.6%)	955 (49.3%)
	1-5	2,075 (49%)	805 (41.7%)
	6-10	970 (22.9%)	150 (7.6%)
	11 or more	150 (3.5%)	25 (1.4%)

For peer review only - http://bmjopen.bmj.com/site/about/guidelines.xhtml

**Clinical Frailty Scale (N, %)**	Unknown	1,520 (35.9%)	150 (7.6%)
	Aged<65	2,030 (48%)	1,735 (89.8%)
	1-3	55 (1.3%)	*
	3-6	245 (5.7%)	25 (1.2%)
	6-9	385 (9.1%)	25 (1.2%)
**Ethnicity (N, %)**	Asian or Asian British	250 (5.9%)	180 (9.2%)
	Black or Black British	60 (1.4%)	50 (2.5%)
	Mixed	35 (0.9%)	30 (1.4%)
	Other Ethnic Groups	50 (1.2%)	65 (3.3%)
	White	3,080 (72.7%)	1,210 (62.7%)
	Unknown	760 (17.9%)	405 (20.9%)
**Deprivation Index (N, %)**	Unknown	420 (10%)	130 (6.6%)
	1-2	1,470 (38.6%)	925 (51.3%)
	3-4	720 (18.9%)	320 (17.7%)
	5-6	655 (17.2%)	245 (13.5%)
	7-8	560 (14.7%)	190 (10.5%)
	9-10	405 (10.6%)	125 (6.9%)

Discussion

Summary

Our study showed that callers with suspected COVID-19 who made an EMS call had a high rate of adverse outcomes (death or organ support) (11.1%, 95% CI: 10.5 to 11.7%). This is around four times greater than the adverse outcome rate in callers who contacted the NHS 111 telephone service and half that seen in an ED population with suspected COVID-19.[18, 21] Ambulances were dispatched to the majority of callers (84.2%) and the decision to dispatch an ambulance achieved a sensitivity of 95%(95% CI: 93.7 to 96.1%) for the primary outcome (death or organ support). Callers for whom an ambulance was not dispatched had a 3.5% or around 1/29 (NPP 96.5% 95% CI: 95.6 to 97.2%) risk of the primary composite adverse outcome. This equated to 70 callers who died or required organ support up to 30 days from first call, not initially being dispatched an ambulance. Our evaluation cannot account for instances where ambulance dispatch was not clinically appropriate despite a high-risk of significant adverse outcomes (e.g. transfer of care to community services for palliative care).

The cost of the high sensitivity achieved by dispatching ambulances to 84% of callers was a low specificity to the primary outcome (death or organ support) (17.2% 95% CI: 16.5% to 17.9%). In total, 33.4% of the cohort had an ambulance dispatched, and were not subsequently conveyed to hospital and nor did they experience the primary outcome. We used multivariable analysis to identify predictors of false negative and false positive triage. The findings need cautious interpretation, given the limited information available during telephone triage, but suggest that some co-morbidities

For peer review only - http://bmjopen.bmj.com/site/about/guidelines.xhtml

For peer review only

(such as chronic respiratory disease) and increasing age may be over-estimated as predictors of adverse outcome.

Strengths and limitations

Although specific EMS telephone triage protocols have been introduced for patients with suspected COVID-19, this appears to be the first evaluation of triage accuracy.[4] Our study used a large cohort of patients identified from routinely collected EMS records and linked this to nationally collected, patient-level healthcare data to provide robust clinical outcomes. We have assessed performance in a cohort of patients with suspected infection which, in the absence of accurate universally available rapid COVID-19 diagnostic tests, reflects the population who must be clinically triaged by urgent and emergency care services.

We have evaluated the performance EMS telephone triage for patients with suspected COVID-19 implemented by the Yorkshire Ambulance Service NHS Trust. Although use of the Card 36 protocol was recommended nationally, there was variation in implementation between different ambulance services.[4] Our study used data from the first wave of the pandemic and it was not until later waves that some ambulance services came under significant pressures due to increased demand.[1] Differences in EMS telephone triage demand and population characteristics of callers with suspected COVID-19 in later waves of the pandemic may affect the estimated accuracy of EMS decision to dispatch an ambulance. The implementation of a senior clinical support model within the call centre, and the influence of this secondary triage both of incoming calls and to support on-scene ambulance decision making was not quantified in this study. It was not possible to identify within the dataset which of the cohort were reviewed by a senior clinician at the initial call.

Our study used routinely collected data and consequently there were high rates of missing data for some variables, such as ethnicity and frailty. This prevented inclusion of these and non-routinely collected variables in analysis. We have assumed that if comorbidities or medication use was not recorded, they were not present. We also assumed in multivariable analysis, patients under the age of 65 had a functional level equivalent to a mild frailty category (CFS 1-3). The mechanism of how data are collected and recorded in the routine data sets used means that there may be bias in the classification of patients. We have previously identified this for the obesity variable where the estimated prevalence of obesity in our cohort is 15% (half that reported in the national health survey) and an implausible protective association with adverse outcomes was observed.[17, 18] As weight is not comprehensively and consistently measured by GPs, estimated statistical effects may reflect unknown characteristics associated with a measurement being taken, rather than the variable itself.

For peer review only - http://bmjopen.bmj.com/site/about/guidelines.xhtml

Implications

EMS telephone triage of ambulance dispatch achieved a higher sensitivity for adverse outcomes (death or organ support) than triage methods used for patient acuity in the ED and NHS-111 COVID-19 assessment pathways.[22, 23] Our cohort had a baseline risk of 11.1% of the primary outcome (death or organ support). The EMS telephone triage system selected those with a lower, 3.5% risk of the primary outcome (death or organ support), not to have an ambulance dispatched. Given the high baseline risk, an alternative would have been to dispatch an ambulance to every caller. This would have led to around 2000 more ambulance being dispatched within the time-period (an 18.8% increase). The acceptable risk of deterioration following telephone triage is subjective and significant variation in risk tolerance between clinicians and public representatives has been demonstrated.[24] When discharging patients from the ED with chest pain, an acceptable risk of a subsequent major adverse cardiovascular events has been found to be 1% or less for the majority of ED clinicians.[25] The risk of false negative triage (3.5%) in our cohort may therefore be unacceptably high for some clinicians. However, this is within the context of a face-to-face assessment with the availability of hospital investigations where greater accuracy may be expected.

Even using EMS telephone triage of ambulance dispatch, around a third of ambulances were dispatched to callers who were not subsequently conveyed to hospital and did not experience the primary outcome. In later waves of the pandemic, EMS providers in the UK experienced significant increases in demand with several ambulance services declaring major incidents.[8, 26, 27] The accuracy of triage observed in our cohort may be the best that realistically can be achieved given the limited information available with telephone triage. However, any measures which can increase accuracy of EMS telephone triage are greatly needed.

Our exploratory multivariable analysis indicates that older age and presence of pre-existing respiratory disease were associated with a higher rate of false positive triage among those who did not have an adverse event (death or organ support), and therefore may be overestimated in the triage process as predictors of adverse outcomes. Being aged 65 years or over is included as a specific high-risk factor in the Card 36 pandemic triage tool.[28] Use of a higher age threshold, or age in conjunction with variables related to performance status, could improve the specificity of EMS ambulance dispatch to significant adverse outcomes.[13] Vaccination against COVID-19 or previous infection may also act to reduce the risk of serious adverse outcomes in suspected infection and these factors are not included in Card 36 assessment.[29]

Limited information is available during EMS telephone triage determining whether ambulance dispatch is required. The Card 36 pandemic triage tool used for this decision making in patients with

For peer review only - http://bmjopen.bmj.com/site/about/guidelines.xhtml

suspected COVID-19 is consensus based. Empirical research assessing the predictive effect of all available variables is required if accuracy of triage, especially in terms of current over-triage and risk of false-negative triage, is to be improved. Given the limitations of the available routine data we have identified in this study, this is likely to require robust prospective data collection to empirically develop and validate more accurate triage tools. The use of trained, non-clinical call handlers for ambulance dispatch contrasts with other telephone triage services.[30] Other models of assessment could improve accuracy and also require evaluation. The current high levels of demand that ambulance services are experiencing mean that even small gains in accuracy could have a large positive effect on both safety and managing increasing demand.

Conclusion

EMS telephone triage of need for ambulance dispatch in the first wave of the pandemic identified a lower risk population to whom ambulances were not dispatched. They constituted 16% of the cohort and around 2000 more ambulances would have been dispatched if these callers received an EMS response. However, patients to whom an ambulance was not dispatched had a clinically significant risk of death or requiring organ support. As the pandemic has developed into later waves, ambulance services in the UK have come under significant sustained pressure due to increased demand. Research to improve the accuracy of EMS telephone triage, especially in terms of safety and specificity, is needed. Due to the limitations of available routinely collected data this is likely to require robust prospective data collection.

Competing Interests

No competing financial interests exist.

Funding

CM is a National Institute for Health Research (NIHR) Clinical Lecturer in Emergency Medicine (Grant Number Not Applicable/NA). The PRIEST study was funded by the UK National Institute for Health Research, Health Technology Assessment (HTA) programme (project reference 11/46/07). This publication presents independent research funded by the National Institute for Health Research and University of Sheffield. The views expressed are those of the author(s) and not necessarily those of the University of Sheffield, the NHS, the NIHR or the Department of Health and Social Care.

Authors' contributions

The idea for the study was conceived by SG, JT, TS, FB, PB, and CM. Data processing and linkage was completed by TS and RC. The analyses were completed by CM and MH with specialist statistical

For peer review only - http://bmjopen.bmj.com/site/about/guidelines.xhtml

advice from SG, JT, LS and PB. CM, TS, MH, RC, PB, JT, RP, BT, LS, FB, KB, FH, SM, JP, and SG contributed to interpretation of results, read, and approved the final manuscript.

Data sharing:

The data used for this study are subject to data sharing agreements with NHS Digital and Yorkshire Ambulance Service which prohibits further sharing of individual level data. The data sets used are obtainable from these organisations subject to necessary authorisations and approvals.

Figures:

Figure 1: STROBE flow diagram of selection of study population

For peer review only

References:

1. https://www.hsj.co.uk/emergency-care/thousands-of-londons-999-calls-answered-by-other-regions/7029302.article.
2. https://www.nationalleadership.gov.uk/supporting-the-nhs-in-its-hour-of-need/london-ambulance-service/.
3. Jensen T, Holgersen MG, Jespersen MS, et al. Strategies to Handle Increased Demand in the COVID-19 Crisis: A Coronavirus EMS Support Track and a Web-Based Self-Triage System. *Prehospital Emergency Care.* 2021;25:28-38.
4. Snooks H, Watkins AJ, Bell F, et al. Call volume, triage outcomes, and protocols during the first wave of the COVID-19 pandemic in the United Kingdom: Results of a national survey. *Journal of the American College of Emergency Physicians Open.* 2021;2:e12492.
5. Ferron R, Agarwal G, Cooper R, Munkley D. The effect of COVID-19 on emergency medical service call volumes and patient acuity: a cross-sectional study in Niagara, Ontario. *BMC Emergency Medicine.* 2021;21:39.
6. Charlton K, Limmer M, Moore H. Incidence of emergency calls and out-of-hospital cardiac arrest deaths during the COVID-19 pandemic: findings from a cross-sectional study in a UK ambulance service. *Emergency Medicine Journal.* 2021;38:446.

For peer review only - http://bmjopen.bmj.com/site/about/guidelines.xhtml

7. Blackwell TH, Kaufman JS. Response time effectiveness: comparison of response time and survival in an urban emergency medical services system. *Academic Emergency Medicine.* 2002;9:288-295.

8. https://www.london.gov.uk/press-releases/mayoral/hospitals-at-risk-of-being-overwhelmed-in-capital.

9. https://www.gov.uk/government/publications/covid-19-guidance-for-ambulance-trusts/covid-19-guidance-for-ambulance-trusts.

10. https://www.emergencydispatch.org/in-the-news/covid-19-communications/a332c5d1-0dbf-4354-9a64-8d149f75f15d.

11. Tankel JW, Ratcliffe D, Smith M, et al. Consequences of the emergency response to COVID-19: a whole health care system review in a single city in the United Kingdom. *BMC Emergency Medicine.* 2021;21:55.

12. https://digital.nhs.uk/services/national-data-opt-out.

13. Goodacre S, Thomas B, Sutton L, et al. Derivation and validation of a clinical severity score for acutely ill adults with suspected COVID-19: The PRIEST observational cohort study. *PLoS One.* 2021;16:e0245840.

14. Campbell MJ, Jacques RM, Fotheringham J, Maheswaran R, Nicholl J. Developing a summary hospital mortality index: retrospective analysis in English hospitals over five years. *BMJ.* 2012;344:e1001.

15. Street A, Maynou L, Gilbert T, Stone T, Mason S, Conroy S. The use of linked routine data to optimise calculation of the Hospital Frailty Risk Score on the basis of previous hospital admissions: a retrospective observational cohort study. *The Lancet Healthy Longevity.* 2021;2:e154-e162.

16. Rockwood K, Song X, MacKnight C, et al. A global clinical measure of fitness and frailty in elderly people. *CMAJ.* 2005;173:489-495.

17. https://digital.nhs.uk/data-and-information/publications/statistical/health-survey-for-england/2019.

18. Marincowitz C, Stone T, Bath P, et al. Accuracy of telephone triage for predicting adverse outcome in suspected COVID-19: An observational cohort study. *medRxiv.* 2021:2021.2006.2024.21259441.

19. Hirst E, Irving A, Goodacre S. Patient and public involvement in emergency care research. *Emergency Medicine Journal.* 2016;33:665-670.

20. https://digital.nhs.uk/binaries/content/assets/website-assets/publications/publications-admin-pages/related-documents/disclosure-control-procedure.pdf.

21. Goodacre S, Thomas B, Lee E, et al. Characterisation of 22445 patients attending UK emergency departments with suspected COVID-19 infection: Observational cohort study. *PLOS ONE.* 2020;15:e0240206.

22. Zachariasse JM, van der Hagen V, Seiger N, Mackway-Jones K, van Veen M, Moll HA. Performance of triage systems in emergency care: a systematic review and meta-analysis. *BMJ Open.* 2019;9:e026471.

23. Thomas B, Goodacre S, Lee E, et al. Prognostic accuracy of emergency department triage tools for adults with suspected COVID-19: the PRIEST observational cohort study. *Emergency Medicine Journal.* 2021:emermed-2020-210783.

24. O'Keeffe ST. A cross-sectional study of doctors', managers' and public representatives' views regarding acceptable level of risk in discharges from the emergency department. *Qjm.* 2015;108:533-538.

25. Than M, Herbert M, Flaws D, et al. What is an acceptable risk of major adverse cardiac event in chest pain patients soon after discharge from the Emergency Department?: A clinical survey. *International Journal of Cardiology.* 2013;166:752-754.

26. https://www.yorkpress.co.uk/news/19012031.yorkshire-ambulance-service-declares-major-incident---report/.

For peer review only - http://bmjopen.bmj.com/site/about/guidelines.xhtml

27. https://www.reuters.com/article/us-health-coronavirus-britain-manchester-idUKKBN27I2HK.

28. https://cdn.emergencydispatch.org/iaed/pdf/NAE-Protocol-36-Flu-v13_3.pdf.

29. Abbasi J. Study Suggests Lasting Immunity After COVID-19, With a Big Boost From Vaccination. *JAMA.* 2021;326:376-377.

30. van Ierland Y, van Veen M, Huibers L, Giesen P, Moll HA. Validity of telephone and physical triage in emergency care: The Netherlands Triage System. *Family Practice.* 2011;28:334-341.

For peer review only

For peer review only - http://bmjopen.bmj.com/site/about/guidelines.xhtml

Figure 1: STROBE flow diagram of study population selection (Created by the authors)

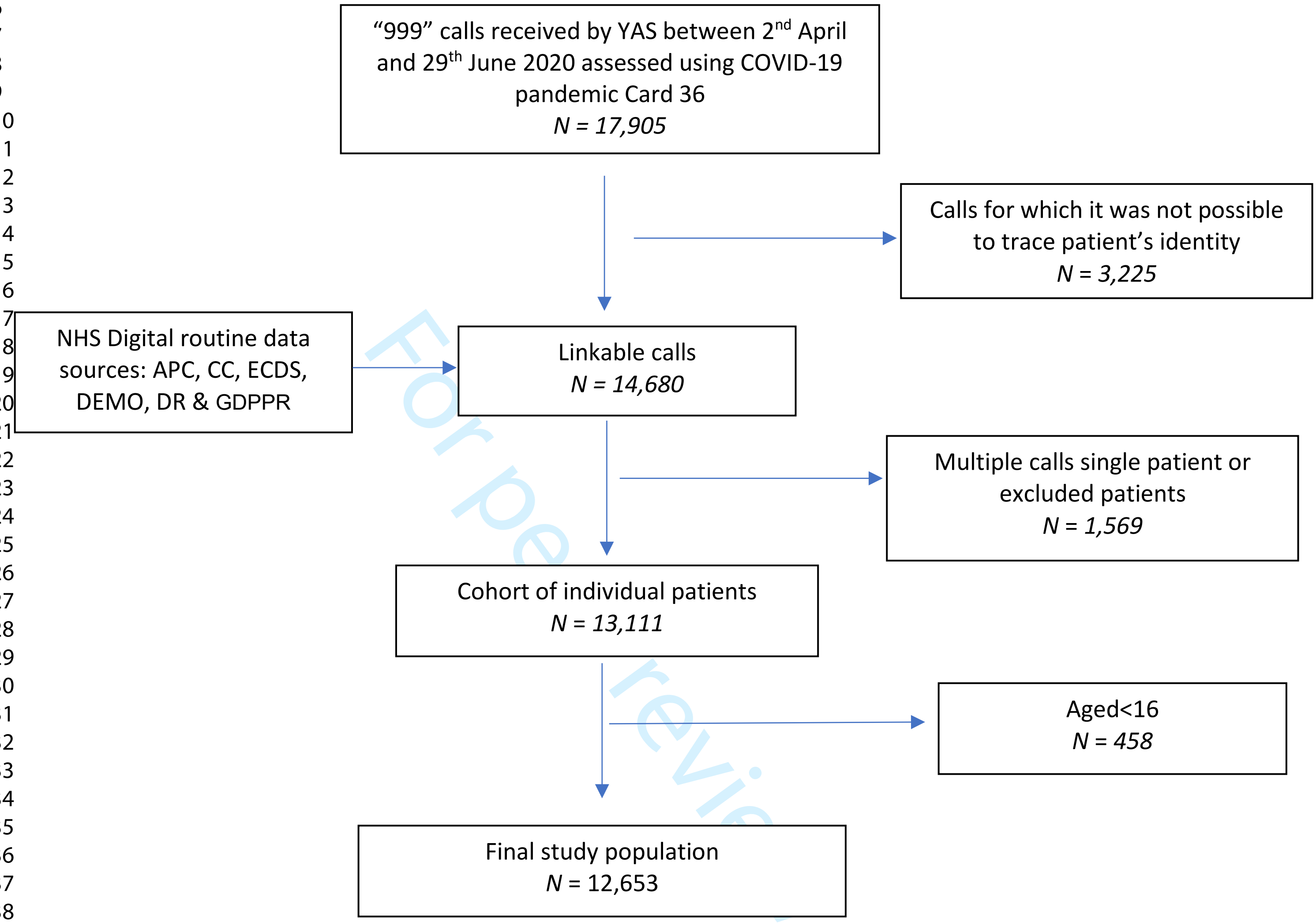

Supplementary Material 1: Summary of AMPDS pandemic Card 36 (Created by the authors) (full details available: https://cdn.emergencydispatch.org/iaed/pdf/resource-library/public%20protocols/pub-protocol%2036/NAE%20CC%2036.pdf)

Questions	Outcome
1) What is the most prominent complaint?	**Delta Category (immediate response)**<br>**Category 1/2 UK Ambulance service response**<br>1) INEFFECTIVE BREATHING with flu-like symptoms<br>2) DIFFICULTY SPEAKING BETWEEN BREATHS with flu-like symptoms<br>3) Not alert with flu-like symptoms<br>4) CHANGING COLOR with flu-like symptoms
If breathing a)Do they have difficulty speaking between breathes?<br>b) Describe their breathing<br>c)Did they have any flu symptoms prior to this?	
If Chest pain a) Are they 35 and over?<br>b) Have they had a heart attack or angina previously	
2) Are they completely alert?	
3) Are they changing colour?	
4) Are they having chills or sweats?	**Charlie Category (Ambulance when able)**<br>**Category 3/4 UK Ambulance service response**<br>1) Abnormal breathing with single flu-like symptom or Asthma/COPD<br>2) Abnormal breathing with multiple flu-like symptoms<br>3) Chest pain/discomfort ≥35 with single flu-like symptom<br>4) Chest pain/discomfort ≥35 with multiple flu-like symptoms<br>5) High Risk Factor
5) Are they vomiting?	
6)Do they have a new cough that recently started?	
7)Do they have a sore throat?	
8)Do they have unusual total body aches?	
9)Do they have a fever?	**Alpha Category (No ambulance dispatched)**<br>1) Chest pain/discomfort < 35 with single flu-like symptom<br>2) Chest pain/discomfort < 35 with multiple flu-like symptoms<br>3) Flu-like symptoms only
10)Do they have a runny or snotty nose?	
11)Do they have diarrhoea?	
12)Do they have a headache?	
13)Do they have any high-risk conditions?<br>• ≤ 5 years old (not COVID-19)<br>• ≥ 65 years old<br>• Blood disorders<br>• Diabetes<br>• Kidney and liver diseases/disorders<br>• Neurological diseases<br>• Pregnancy (up to 2 weeks after delivery)<br>• Sickle cell disease (sickle cell anaemia)<br>• Weakened immune system	

Supplementary Material 2: Multi-variable model predicting false negatives (Created by the authors)

Population Characteristic	Level	Odds ratio (95% CI) N= 1215
**Age (Years)**	1-year increase	0.95 (0.94 to 0.97)
**Sex**	Female	1.89 (1.09 to 3.26)
**Comorbidity**	Cardiovascular Disease	0.73 (0.21 to 2.56)
	Chronic Resp. Disease	0.50 (0.23 to 1.09)
	Diabetes	1.19 (0.60 to 2.38)
	Hypertension	0.75 (0.39 to 1.46)
	Immunosuppression (including steroid use)	0.73 (0.35 to 1.53)
	Active Malignancy	0.12 (0.02 to 0.92)
	Obesity	Not included
	Renal Impairment	2.22 (0.71 to 6.96)
	Smoker	0.96 (0.52 to 1.80)
	Stroke	1.93 (0.42 to 8.80)
**Number of Drugs Used**	0	Reference
	1-5	0.98 (0.45 to 2.15)
	6-10	1.56 (0.55 to 4.44)
	11 or more	0.76 (0.08 to 7.42)
**Deprivation Index**	1-2	Reference
	3-4	0.98 (0.47 to 2.08)
	5-6	0.54 (0.21 to 1.36)
	7-8	1.37 (0.64 to 2.97)
	9-10	0.99 (0.38 to 2.59)

For peer review only

Supplementary Material 3: Multi-variable model predicting false positives

Population Characteristic	Level	Odds ratio (95% CI)<br>N= 5, 615
**Age (Years)**	1-year increase	1.05 (1.04 to 1.05)
**Sex**	Female	1.31 (1.15 to 1.49)
**Comorbidity**	Cardiovascular Disease	1.38 (0.85 to 2.24)
	Chronic Resp. Disease	1.35 (1.13 to 1.60)
	Diabetes	0.85 (0.67 to 1.09)
	Hypertension	1.03 (0.84 to 1.26)
	Immunosuppression (including steroid use)	1.02 (0.82 to 1.26)
	Active Malignancy	1.25 (0.72 to 2.18)
	Renal Impairment	0.80 (0.44 to 1.44)
	Smoker	0.86 (0.75 to 0.99)
	Stroke	1.17 (0.59 to 2.31)
**Number of Drugs Used**	0	Reference
	1-5	0.99 (0.84 to 1.16)
	6-10	1.22 (0.90 to 1.66)
	11 or more	1.13 (0.62 to 2.06)
**Deprivation Index**	1-2	Reference
	3-4	1.21 (1.02 to 1.44)
	5-6	1.23 (1.01 to 1.48)
	7-8	1.29 (1.05 to 1.59)
	9-10	1.32 (1.04 to 1.69)

For peer review only

The RECORD statement – checklist of items, extended from the STROBE statement, that should be reported in observational studies using routinely collected health data.

	Item No.	STROBE items	Location in manuscript where items are reported	RECORD items	Location in manuscript where items are reported
**Title and abstract**					
	1	(a) Indicate the study's design with a commonly used term in the title or the abstract (b) Provide in the abstract an informative and balanced summary of what was done and what was found	Pages 1-2	RECORD 1.1: The type of data used should be specified in the title or abstract. When possible, the name of the databases used should be included.<br><br>RECORD 1.2: If applicable, the geographic region and timeframe within which the study took place should be reported in the title or abstract.<br><br>RECORD 1.3: If linkage between databases was conducted for the study, this should be clearly stated in the title or abstract.	Page 2 (abstract)
**Introduction**					
Background rationale	2	Explain the scientific background and rationale for the investigation being reported	Page 3		
Objectives	3	State specific objectives, including any prespecified hypotheses	Page 3		
**Methods**					
Study Design	4	Present key elements of study design early in the paper	Page 4		
Setting	5	Describe the setting, locations, and relevant dates, including periods of recruitment, exposure, follow-up, and data collection	Page 4		

For peer review only

Participants	6	*(a) Cohort study* - Give the eligibility criteria, and the sources and methods of selection of participants. Describe methods of follow-up<br>*Case-control study* - Give the eligibility criteria, and the sources and methods of case ascertainment and control selection. Give the rationale for the choice of cases and controls<br>*Cross-sectional study* - Give the eligibility criteria, and the sources and methods of selection of participants<br><br>*(b) Cohort study* - For matched studies, give matching criteria and number of exposed and unexposed<br>*Case-control study* - For matched studies, give matching criteria and the number of controls per case	Page 4,5	RECORD 6.1: The methods of study population selection (such as codes or algorithms used to identify subjects) should be listed in detail. If this is not possible, an explanation should be provided.<br><br>RECORD 6.2: Any validation studies of the codes or algorithms used to select the population should be referenced. If validation was conducted for this study and not published elsewhere, detailed methods and results should be provided.<br><br>RECORD 6.3: If the study involved linkage of databases, consider use of a flow diagram or other graphical display to demonstrate the data linkage process, including the number of individuals with linked data at each stage.	Page 4,5<br>Figure 1
Variables	7	Clearly define all outcomes, exposures, predictors, potential confounders, and effect modifiers. Give diagnostic criteria, if applicable.	Page 5,6	RECORD 7.1: A complete list of codes and algorithms used to classify exposures, outcomes, confounders, and effect modifiers should be provided. If these cannot be reported, an explanation should be provided.	
Data sources/ measurement	8	For each variable of interest, give sources of data and details of methods of assessment (measurement).<br>Describe comparability of assessment methods if there is more than one group	Page 5,6		

For peer review only

Bias	9	Describe any efforts to address potential sources of bias	Page 5-6		
Study size	10	Explain how the study size was arrived at	Pages 4,5 Figure 1		
Quantitative variables	11	Explain how quantitative variables were handled in the analyses. If applicable, describe which groupings were chosen, and why	Pages 5,6		
Statistical methods	12	(a) Describe all statistical methods, including those used to control for confounding<br>(b) Describe any methods used to examine subgroups and interactions<br>(c) Explain how missing data were addressed<br>(d) *Cohort study* - If applicable, explain how loss to follow-up was addressed<br>*Case-control study* - If applicable, explain how matching of cases and controls was addressed<br>*Cross-sectional study* - If applicable, describe analytical methods taking account of sampling strategy<br>(e) Describe any sensitivity analyses	Pages 5,6		
Data access and cleaning methods		..	Page 4	RECORD 12.1: Authors should describe the extent to which the investigators had access to the database population used to create the study population.	Page 4

For peer review only

				RECORD 12.2: Authors should provide information on the data cleaning methods used in the study.	
Linkage		..	Page 4	RECORD 12.3: State whether the study included person-level, institutional-level, or other data linkage across two or more databases. The methods of linkage and methods of linkage quality evaluation should be provided.	Figure 1
**Results**					
Participants	13	(a) Report the numbers of individuals at each stage of the study (*e.g.*, numbers potentially eligible, examined for eligibility, confirmed eligible, included in the study, completing follow-up, and analysed)<br>(b) Give reasons for non-participation at each stage.<br>(c) Consider use of a flow diagram	Figure 1	RECORD 13.1: Describe in detail the selection of the persons included in the study (*i.e.,* study population selection) including filtering based on data quality, data availability and linkage. The selection of included persons can be described in the text and/or by means of the study flow diagram.	Figure 1
Descriptive data	14	(a) Give characteristics of study participants (*e.g.*, demographic, clinical, social) and information on exposures and potential confounders<br>(b) Indicate the number of participants with missing data for each variable of interest<br>(c) *Cohort study* - summarise follow-up time (*e.g.*, average and total amount)	Table 1		
Outcome data	15	*Cohort study* - Report numbers of outcome events or summary measures over time<br>*Case-control study* - Report numbers in each exposure	Page 6-8		

For peer review only

		category, or summary measures of exposure<br>*Cross-sectional study* - Report numbers of outcome events or summary measures			
Main results	16	(a) Give unadjusted estimates and, if applicable, confounder-adjusted estimates and their precision (e.g., 95% confidence interval). Make clear which confounders were adjusted for and why they were included<br>(b) Report category boundaries when continuous variables were categorized<br>(c) If relevant, consider translating estimates of relative risk into absolute risk for a meaningful time period	Pages 8-11		
Other analyses	17	Report other analyses done—e.g., analyses of subgroups and interactions, and sensitivity analyses	Pages 8-11		
**Discussion**					
Key results	18	Summarise key results with reference to study objectives	Pages 12		
Limitations	19	Discuss limitations of the study, taking into account sources of potential bias or imprecision. Discuss both direction and magnitude of any potential bias	Pages 12-13	RECORD 19.1: Discuss the implications of using data that were not created or collected to answer the specific research question(s). Include discussion of misclassification bias, unmeasured confounding, missing data, and changing eligibility over time, as they pertain to the study being reported.	Pages 12-13
Interpretation	20	Give a cautious overall interpretation of results considering objectives,	Pages 13-14		

For peer review only

		limitations, multiplicity of analyses, results from similar studies, and other relevant evidence			
Generalisability	21	Discuss the generalisability (external validity) of the study results	Pages 12-14		
**Other Information**					
Funding	22	Give the source of funding and the role of the funders for the present study and, if applicable, for the original study on which the present article is based	15		
Accessibility of protocol, raw data, and programming code		..	15	RECORD 22.1: Authors should provide information on how to access any supplemental information such as the study protocol, raw data, or programming code.	15

*Reference: Benchimol EI, Smeeth L, Guttmann A, Harron K, Moher D, Petersen I, Sørensen HT, von Elm E, Langan SM, the RECORD Working Committee. The REporting of studies Conducted using Observational Routinely-collected health Data (RECORD) Statement. *PLoS Medicine* 2015; in press.

*Checklist is protected under Creative Commons Attribution (CC BY) license.

For peer review only

BMJ Open

Accuracy of emergency medical service telephone triage of need for an ambulance response in suspected COVID-19: An observational cohort study

Journal:	*BMJ Open*
Manuscript ID	bmjopen-2021-058628.R2
Article Type:	Original research
Date Submitted by the Author:	13-Apr-2022
Complete List of Authors:	Marincowitz, Carl; The University of Sheffield, Centre for Urgent and Emergency Care Research<br>Stone, Tony; The University of Sheffield, Centre for Urgent and Emergency Care Research<br>Hasan, Madina ; The University of Sheffield, Centre for Urgent and Emergency Care Research<br>Campbell, Richard; The University of Sheffield, Centre for Urgent and Emergency Care Research<br>Bath, Peter; The University of Sheffield, Information School<br>Turner, Janette; The University of Sheffield, Centre for Urgent and Emergency Care Research<br>Pilbery, Richard; Yorkshire Ambulance Service NHS Trust, Research and Development<br>Thomas, Benjamin; The University of Sheffield, Clinical Trials Research Unit<br>Sutton, Laura ; The University of Sheffield, Clinical Trials Research Unit<br>Bell, Fiona; Yorkshire Ambulance Service NHS Trust, Research and Development<br>Biggs, Katie; The University of Sheffield, Clinical Trials Research Unit<br>Hopfgartner, Frank; The University of Sheffield, Information School<br>Mazumdar, Suvodeep; The University of Sheffield, Information School<br>Petrie, Jennifer; The University of Sheffield, Clinical Trials Research Unit<br>Goodacre, Steve; The University of Sheffield, Centre for Urgent and Emergency Care Research
**Primary Subject Heading**:	Emergency medicine
Secondary Subject Heading:	Respiratory medicine, Health services research
Keywords:	COVID-19, Risk management < HEALTH SERVICES ADMINISTRATION & MANAGEMENT, ACCIDENT & EMERGENCY MEDICINE

SCHOLARONE™
Manuscripts

I, the Submitting Author has the right to grant and does grant on behalf of all authors of the Work (as defined in the below author licence), an exclusive licence and/or a non-exclusive licence for contributions from authors who are: i) UK Crown employees; ii) where BMJ has agreed a CC-BY licence shall apply, and/or iii) in accordance with the terms applicable for US Federal Government officers or employees acting as part of their official duties; on a worldwide, perpetual, irrevocable, royalty-free basis to BMJ Publishing Group Ltd ("BMJ") its licensees and where the relevant Journal is co-owned by BMJ to the co-owners of the Journal, to publish the Work in this journal and any other BMJ products and to exploit all rights, as set out in our licence.

The Submitting Author accepts and understands that any supply made under these terms is made by BMJ to the Submitting Author unless you are acting as an employee on behalf of your employer or a postgraduate student of an affiliated institution which is paying any applicable article publishing charge ("APC") for Open Access articles. Where the Submitting Author wishes to make the Work available on an Open Access basis (and intends to pay the relevant APC), the terms of reuse of such Open Access shall be governed by a Creative Commons licence – details of these licences and which Creative Commons licence will apply to this Work are set out in our licence referred to above.

Other than as permitted in any relevant BMJ Author's Self Archiving Policies, I confirm this Work has not been accepted for publication elsewhere, is not being considered for publication elsewhere and does not duplicate material already published. I confirm all authors consent to publication of this Work and authorise the granting of this licence.

For peer review only

Accuracy of emergency medical service telephone triage of need for an ambulance response in suspected COVID-19: An observational cohort study

Carl Marincowitz[1] NIHR Clinical Lecturer Emergency Medicine

Tony Stone[1] Senior Data Manager

Madina Hasan[1] Research Associate

Richard Campbell[1] Data Specialist

Peter A. Bath[1,2] Professor of Health Informatics and Health Data Analytics

Janette Turner[1] Honorary Senior Research Fellow

Richard Pilbery[3] Research paramedic

Benjamin Thomas[4] Study Manager

Laura Sutton[4] Research Associate

Fiona Bell[3] Head of Research

Katie Biggs[4] Research Fellow/Assistant CTRU Director

Frank Hopfgartner[2] Senior Lecturer in Data Science

Suvodeep Mazumdar[2] Lecturer in Data Analytics

Jennifer Petrie[4] Trial Manager

Steve Goodacre[1] Professor of Emergency Medicine

1. Centre for Urgent and Emergency Care Research (CURE), Health Services Research School of Health and Related Research, University of Sheffield, Regent Court, 30 Regent Street, Sheffield, S1 4DA, UK, Fax: +44 (0)114 222 0749 Tel: (+44) (0)114 222 4345, Email: C.Marincowitz@Sheffield.ac.uk
2. Information School, University of Sheffield, Regent Court, 211 Portobello St, Sheffield S1 4DP, UK
3. Yorkshire Ambulance Service NHS Trust, Trust HQ, Springhill 2, Brindley Way, Wakefield, United Kingdom, WF2 0XQ
4. Clinical Trials Research Unit (CTRU), Health Services Research School of Health and Related Research, University of Sheffield, Regent Court, 30 Regent Street, Sheffield, S1 4DA, UK, Fax: +44 (0)114 222 0749 Tel: (+44) (0)114 222 4345,

Correspondence to Dr Benjamin Thomas at b.d.thomas@sheffield.ac.uk

For peer review only - http://bmjopen.bmj.com/site/about/guidelines.xhtml

Abstract

Objective: To assess accuracy of Emergency Medical Service (EMS) telephone triage in identifying patients who need an EMS response and identify factors which affect triage accuracy.

Design: Observational cohort study

Setting: Emergency telephone triage provided by Yorkshire Ambulance Service NHS Trust (YAS).

Participants: 12, 653 adults who contacted EMS telephone triage services provided by YAS between the 2nd April 2020 and 29th June 2020 assessed by COVID-19 telephone triage pathways were included.

Outcome: Accuracy of call handler decision to dispatch an ambulance was assessed in terms of death or need for organ support at 30 days from first contact with the telephone triage service.

Results: Callers contacting EMS dispatch services had an 11.1% (1,405/12,653) risk of death or needing organ support. In total, 1,335/12,653 (16%) of callers did not receive an emergency response and they had a 70/1,335 (3.5%) risk of death or organ support. Ambulances were dispatched to 4, 230 callers (33.4%) who were not conveyed to hospital and did not deteriorate. Multivariable modelling found variables of older age (1 year increase OR 1.05, 95% CI:1.04 to 1.05) and presence of pre-existing respiratory disease (OR 1.35 95% CI:1.13 to 1.60) to be predictors of false positive triage.

Conclusion: Telephone triage can reduce ambulance responses but, with low specificity. A small but significant proportion of patients who do not receive an initial emergency response deteriorated. Research to improve accuracy of EMS telephone triage is needed and, due to limitations of routinely collected data, this is likely to require prospective data collection.

Strengths and limitations of this study

- Despite concerns regarding accuracy in identifying need for emergency treatment, this is one of the first evaluations of EMS telephone triage protocols for suspected COVID-19.
- Use of ambulance data linked to nationally collected death registrations and routinely collected healthcare data provided robust outcome data for all included callers.
- Use of routine data limited assessment of factors which affect triage accuracy to those which are routinely collected.
- The evaluation in this study is of a single United Kingdom (UK) ambulance service's implementation of EMS telephone triage protocols for suspected COVID-19 cases.

Word Count: 3, 248

For peer review only - http://bmjopen.bmj.com/site/about/guidelines.xhtml

Background

During the COVID-19 pandemic, emergency medical service (EMS) call volumes have been volatile in the United Kingdom (UK), Europe and North America. Some ambulance services in the UK reported up to three times the expected number of EMS calls during the first and second waves of the pandemic, an increase also observed in other parts of Europe.[1-4] Other EMS providers in the UK and North America observed initial decreases in call volumes.[5, 6] Surges in demand in the second wave of the pandemic led to some ambulance services in the UK declaring major incidents and warning of care being compromised by overwhelming demand.[7, 8]

EMS call handlers in the UK are typically trained non-clinical staff who use either the Advanced Medical Priority Dispatch System (AMPDS) or NHS Pathways to triage the EMS response to calls. [4] On the 2nd April 2020, six English NHS ambulance services using AMPDS introduced a specific protocol for callers with suspected COVID-19 using the pandemic Card 36.[9, 10] Structured questions (summarised in Supplementary Material 1) were used to triage urgency of EMS response into three levels: Delta (highest priority requiring immediate response), Charlie (medium priority requiring ambulance attendance when able) and Alpha (no ambulance dispatched). The AMPDS Card 36 was implemented by UK ambulance services in different ways, with some symptoms and selected high risk-groups receiving a higher priority response than normal, with alterations to the card implemented in the following months.[4]

There is evidence that changes in EMS practice due to the pandemic and delays in the emergency assessment of patients with COVID-19 may have contributed to avoidable deaths in the North West of England.[11] Despite the need for EMS telephone triage to balance ambulance responses to patients requiring life-saving interventions against an anticipation of overwhelming demand during the pandemic, there has been no previous evaluation of the accuracy of the clinical risk-assessment performed for patients with suspected COVID-19.

Our study aimed to:

1) Assess how accurately EMS telephone triage identified those likely to suffer an adverse outcome (death or organ support) and subsequently require an emergency ambulance response.
2) Identify any factors that may have affected the accuracy of EMS telephone triage.

Methods

Study Design and Setting

For peer review only - http://bmjopen.bmj.com/site/about/guidelines.xhtml

This observational cohort study used linked routinely collected EMS telephone call centre data from Yorkshire Ambulance Service NHS Trust (YAS) to assess the accuracy of clinical triage of patients with suspected COVID-19.

Emergency services provided by YAS covers a region in the north of England of approximately 6,000 square miles and with a population of 5.3 million. In 2020/2021, YAS received more than 1,000,000 emergency (999) calls.

Data Sources and linkage

Yorkshire Ambulance Service provided a dataset of all 999 calls, triaged using a Card 36 pandemic triage pathway for patients with suspected COVID-19, received between the 2nd April and 29th June 2020. The dataset consisted of patient identifiers, demographic data, call details and the outcome of the call (including whether an ambulance was dispatched) extracted from routinely collected electronic call records.

Health and social care data relating to the population in England within the UK National Health Service (NHS) are managed by NHS Digital. We provided patient identifiers to NHS Digital to trace patients in our cohort and supply additional individual-level demographic, co-morbidity and outcome data. NHS Digital identified records in their collections belonging to patients in our cohort, and provided data on patient demographics, limited COVID-related general practice (GP) records, emergency department attendances, hospital inpatient admissions, critical care periods, and death registrations from the UK Office for National Statistics (ONS).

YAS and NHS Digital removed records where patients indicated that they did not wish their data to be used for research purposes, via the NHS data opt-out service.[12] The study team also excluded patients who had opted out of any part of the wider PRIEST study,[13] of which this evaluation forms a part, and those with inconsistent records (e.g., multiple deaths recorded or death before latest activity). Patient identifiers across all datasets were replaced with a consistent pseudo-identifier to enable the identification and linkage of records belonging to the same patient across all datasets but without revealing any patient identifiers. Calls which originated from other health care services (i.e., where a decision that ambulance dispatch was required had already been made) were excluded from the study population.

Inclusion criteria

Our final cohort consisted of all adult (aged 16 years and over) callers at time of first (index) EMS 999 call between 2nd April and 29th June 2020 assessed using the suspected COVID-19 Card 36 triage pathway, who were successfully traced by NHS Digital.

For peer review only - http://bmjopen.bmj.com/site/about/guidelines.xhtml

Outcome

The primary outcome was death, renal, respiratory, or cardiovascular organ support (identified from death registration and critical care data) at 30 days from index contact.

The secondary outcomes were hospital conveyance following ambulance dispatch (transfer to ED or inpatient setting) and inpatient admission (recoded inpatient dataset) 30 days from index contact. The 30-day duration of outcomes was based on the duration used when deriving the PRIEST clinical severity score.[13]

Patient Characteristics

Consistent with methods used to estimate the Charlson comorbidity index from the available routine data, comorbidities were included if recorded within 12 months before the index EMS call.[14, 15] In a similar way, only immunosuppressant drug prescriptions documented in GP records within 30 days before the index contact, contributed to the immunosuppression co-morbidity variable. Pregnancy status was based on GP records recorded in the previous 9 months. Frailty in patients older than 65 years was derived from the latest recorded Clinical Frailty Scale (CFS) score (if recorded) in the electronic GP records prior to index attendance.[16] Patients under the age of 65 years were not given a CFS score since it is not validated in this age group. However, in multivariable analysis, patients under the age of 65 were assumed to have a functional level equivalent to a mild frailty category (CFS 1-3).

Analysis

We conducted a descriptive analysis of caller demographics, co-morbidities and call disposition. The proportion of callers who experienced the primary and secondary outcome was estimated. To assess the accuracy of EMS telephone triage in identifying clinical outcomes requiring an emergency response, the call disposition of the index contact was divided into the binary classification of either: ambulance dispatched (Delta or Charlie priority); or other call outcome (Alpha priority) (Supplementary Material 1). We assessed the accuracy of the binary triage classification (ambulance dispatched versus no ambulance dispatched) in terms of sensitivity, specificity, positive predictive value (PPV) and negative predictive value (NPV) for the primary and secondary outcomes with 95% confidence intervals (CI).

Patient characteristics of false negatives (those who experienced the primary outcome (death or organ support) and no ambulance was dispatched) and true positives (ambulance dispatched who

For peer review only - http://bmjopen.bmj.com/site/about/guidelines.xhtml

experienced the primary outcome) were compared. In patients with the adverse outcome (death or organ support) multi-variable logistic regression was used to identify patient characteristics associated with false negative triage. We also compared the characteristics of false positives (ambulance dispatched and not conveyed to hospital) and true negatives (no ambulance dispatched) among those who did not experience the primary composite outcome. We used multivariable logistic regression to identify factors which predicted false positive EMS triage. Obesity was excluded from multivariable analysis due to an observed implausible protective association with the primary outcome (death or organ support), which we believe to be an artefact of how these data were collected and recorded in the electronic GP data set.[17, 18] Ethnicity and frailty were also excluded from multivariable analysis due to the high proportion of missing data. All analyses were completed using STATA 16 (StataCorp. 2019. *Stata Statistical Software: Release 16*. College Station, TX: StataCorp LLC).

Ethical Approval

The North West—Haydock Research Ethics Committee gave a favourable opinion on the PAINTED study on 25th June 2012 (reference 12/NW/0303) and on the updated PRIEST study on 23rd March 2020, including the analyses presented here. The Confidentiality Advisory Group of the NHS Health Research Authority granted approval to collect data without patient consent in line with Section 251 of the National Health Service Act 2006. Access to data collected by NHS Digital was recommended for approval by its Independent Group Advising on the Release of Data (IGARD) on 11th September 2021, having received additional recommendation for approval for access to GP records from the Profession Advisory Group (PAG) on 19th August 2021.

Patient Public Involvement

The Sheffield Emergency Care Forum (SECF) is a public representative group interested in emergency care research.[19] Members of SECF advised on the development of the PRIEST study and two members joined the Study Steering Committee. Patients were not involved in the conduct of the study.

Results

All totals (including outcome) presented are rounded to the nearest 5, with small numbers suppressed to comply with NHS Digital data disclosure guidance.[20]

For peer review only - http://bmjopen.bmj.com/site/about/guidelines.xhtml

For peer review only

Study population

Figure 1 and Table 1 summarise study cohort derivation and the characteristics of the 12, 653 included individual callers. In total, 1,405 callers (11.1%, 95% CI: 10.5 to 11.7%) experienced the primary outcome (death or organ support) within 30 days following the index EMS 999 call. An ambulance was dispatched to 10,650 (84.2%) of callers. In our study cohort, 6,070 patients (48%. 95%: 47.1 to 48.9%) were conveyed to hospital (ED or directly to an inpatient setting) and 5,735 (45.3%, 95% CI: 44.5 to 46.2%) were admitted as hospital inpatients within 30 days of index contact.

The median age of the whole cohort was 66 (IQR = 46-81), the cohort had an almost equal proportion of males (49.5%) and females (50.5%) and had high rates of comorbidity (chronic respiratory disease 29.5%, diabetes 16.6% and hypertension 35.9%).

Table 1: Population characteristics

Population Characteristic	Level	Whole Population N=12, 653	No Death or organ support (30 days)* N= 11,250	Death or organ support(30 days)* N=1,405
**Age (Years)**	Median (IQR)**<br>Mean	66 (46-81)<br>62.3	63 (43-79)<br>60.6	80 (67-87)<br>70
**Sex (N, %)***	Male	6,260 (49.5%)	5,465 (48.6%)	795 (56.5%)
**Comorbidity (N, %)***	Cardiovascular Disease	810 (6.4%)	690 (6.1%)	120 (8.6%)
	Chronic Resp. Disease	3,730 (29.5%)	3,320 (29.5%)	410 (29.4%)
	Diabetes	2,095 (16.5%)	1,770 (10.2%)	325 (23.1%)
	Hypertension	4,545 (35.9%)	6,760 (15.7%)	705 (50.4%)
	Immunosuppression (including steroid use)	2, 540 (20.1%)	3,545 (31.5%)	360 (25.8%)
	Active Malignancy	595 (4.7%)	435 (3.9%)	160 (11.5%)
	Obesity	1,960 (15.5%)	1,860 (16.5%)	100 (7.3%)
	Pregnant	135 (1.1%)	135 (1.2%)	-
	Renal Impairment	365 (2.9%)	310 (2.7%)	60 (4.3%)
	Stroke	285 (2.2%)	240 (2.1%)	45 (3.1%)
**Social (N, %)***	Smoker	4,810 (38.0%)	1,740 (15.5%)	550 (39.3%)
**Number of Prescribed Drugs Used***	0	3,115 (24.6%)	2,940 (15.1)	175 (12.4%)
	1-5	6,015 (47.5%)	5,320 (47.3%)	700 (49.8%)
	6-10	3,030 (23.9%)	2,565 (22.8%)	465 (33.1%)

For peer review only - http://bmjopen.bmj.com/site/about/guidelines.xhtml

**(N, %)**	11 or more	490 (3.9%)	425 (3.8%)	65 (4.7%)
**Clinical Frailty Scale (N, %)***	Unknown	4,560 (36.0%)	3,875 (34.4%)	685 (48.9%)
	Aged<65	6,105 (48.2%)	5,800 (51.5%)	305 (21.6%)
	1-3	165 (1.3%)	150 (1.3%)	15 (1.0%)
	3-6	670 (5.3%)	590 (5.2%)	85 (6.0%)
	6-9	1,155 (9.1%)	840 (7.5%)	315 (22.5%)
**Ethnicity (N, %)***	Asian or Asian British	750 (5.9%)	700 (6.2%)	5 (3.6%)
	Black or Black British	190 (1.5%)	180 (1.6%)	15 (0.9%)
	Mixed	110 (0.9%)	105 (0.9%)	-
	Other Ethnic Groups	160 (1.3%)	150 (1.3%)	-
	White	9,020 (71.3%)	8,070 (71.7%)	950 (67.8%)
	Unknown	2,425 (19.2%)	2,050 (18.2%)	375 (26.6%)
**Deprivation Index (N, %)***	Unknown	1,250 (9.9%)	1,060 (9.4%)	190 (13.5%)
	1-2	4,600 (40.3%)	4,155 (40.8%)	440 (36.4%)
	3-4	2,075 (18.2%)	1,855 (18.2%)	220 (18%)
	5-6	1,880 (16.5%)	1,660 (16.3%)	220 (18%)
	7-8	1,695 (14.9%)	1,485 (14.6%)	210 (17.2%)
	9-10	1,160 (10.2%)	1,030 (10.1%)	130 (10.6%)
**Ambulance Dispatched (N, %)***	Ambulance	10,650 (84.2%)	9,320 (82.8%)	1,335 (95%)
	No Ambulance	2,000 (15.8%)	1,930 (17.2%)	70 (5%)
**Outcome (N, %)***	Death	1,155 (9.1%)	NA***	1,155 (82.4%)
	Deaths due to COVID (including after 30 days)	690 (5.5%)	NA	530 (37.8%)
	Organ support (within 30 days)	335 (2.6%)	NA	335 (23.8%)
**Hospitalisation (N, %)***	ED Attendance	6,945 (54.9%)	5,975 (53.1%)	970 (69.1%)
	Inpatient admission	5,735 (45.3%)	4,650 (41.3%)	1,085 (77.3%)
**Confirmed Hospital Diagnosis of COVID (N, %)*□**	In ED or as inpatient at 30 days	1,895 (15%)	1,300 (11.6%)	595 (42.3%)
**Time to Primary Outcome from index contact- up to and including (N, %)**	72 hours	475 (3.8%)	NA	475 (33.6%)
	7 days	780 (6.1%)	**NA**	780 (55.5%)

*To comply with NHS digital disclosure guidance totals for these variables are rounded to the nearest 5, which may result in apparent disparities in the overall totals

**Interquartile Range (IQR)

*** Not Applicable (NA)

□Unrestricted community testing for suspected COVID-19 was only available from the 18/05/2020. Confirmed diagnosis is based upon inpatient PCR testing or clinical diagnosis in hospital

- Suppressed due to small numbers

For peer review only - http://bmjopen.bmj.com/site/about/guidelines.xhtml

Accuracy of telephone triage of ambulance dispatch

Table 2 shows the accuracy of the EMS telephone triage decision to dispatch an ambulance for the composite primary outcome (death or organ support) indicating need for emergency intervention. Decision to dispatch an ambulance achieved a sensitivity of 95% (95% CI:93.7 to 96.1%) to the primary outcome. If advised to self-care/non-urgent clinical assessment, the chance of experiencing an adverse outcome (death or organ support) was approximately 3.5% (NPV: 96.5%, 95% CI: 95.6 to 97.2%). The high sensitivity was achieved at the cost of specificity (17.2% 95% CI: 16.5% to 17.9%). Among patients for whom an ambulance was dispatched, the risk of serious adverse outcomes was 12.5% (95% CI: 11.9 to 13.2%), transfer to hospital was 57% (95% CI: 56 to 58%) and hospital admission 49.3% (48.3 to 50.2%).

Table 2: Performance of decision to dispatch ambulance for composite primary outcome (death or organ support)

Primary outcome 30 days (11.1%, 10.5-11.7%)			
N= 12,653	**Death or organ support**	**No death or organ support**	
Ambulance dispatched	1,335	9,320	Sensitivity 95% (93.7-96.1%)<br>Positive Predictive Value 12.5% (11.9 – 13.2%)
No Ambulance dispatched	70	1,930	Specificity 17.2% (16.5% - 17.9%)<br>Negative Predictive Value 96.5% (95.6 - 97.2%)

Prediction of false negative or false positive ambulance dispatch

Table 3 compares the characteristics of patients who experienced the primary outcome (death or organ support), and either did (true positives) or did not (false negatives) have an ambulance dispatched on index call. In both groups, over 50% of people experienced the primary adverse outcome within seven days of first contact and around 33% of patients experienced the adverse outcome within 72 hours of index assessment. Multivariable modelling (Supplementary Material 2) showed that female sex (OR 1.89, 95% CI 1.09 to 3.26) was associated with increased risk and,

For peer review only - http://bmjopen.bmj.com/site/about/guidelines.xhtml

increasing age (OR 0.95, 95% CI 0.94 to 0.97) and malignancy (OR 0.12, 95% CI 0.02 to 0.92) reduced risk, of false negative triage.

Table 3: False negatives compared to true positives

Population Characteristic	Level	False Negatives (No ambulance dispatch and primary outcome 30 days) N= 70	True Positives (Ambulance dispatch and primary outcome 30 days) N= 1,335
Age (Years)	Median (IQR)<br>Mean	61.5 (51-80)<br>64	80 (68-87)<br>76.7
Sex (N, %)	Male	35 (51.4%)	760 (56.8%)
Comorbidity (N, %)	Cardiovascular Disease	*	115 (8.8%)
	Chronic Resp. Disease	15 (20%)	400 (29.9%)
	Diabetes	20 (25.7%)	305 (23%)
	Hypertension	25 (34.3%)	685 (51.2%)
	Immunosuppression (including steroid use)	10 (17.1%)	350 (26.3%)
	Active Malignancy	*	160 (11.9%)
	Obesity	10 (12.9%)	95 (7%)
	Pregnant	*	*
	Renal Impairment	*	55 (4.2%)
	Stroke	*	40 (3.2%)
Social (N, %)	Smoker	25 (32.9%)	530 (39.6%)
Number of Drugs Used (N, %)	0	20 (25.7%)	155 (11.7%)
	1-5	30 (42.9%)	670 (50.2%)
	6-10	20 (28.6%)	445 (33.3%)
	11 or more	*	65 (4.8%)
Clinical Frailty Scale (N, %)	Unknown	60 (84.3%)	665 (49.8%)
	Aged<65	*	260 (19.7%)
	1-3	*	15 (1%)
	3-6	*	80 (6.2%)
	6-9	*	310 (23.4%)
Ethnicity (N, %)	Asian or Asian British	*	45 (3.5%)
	Black or Black British	*	15 (1%)
	Mixed	*	*
	Other Ethnic Groups	*	10 (0.7%)
	White	45 (61.4%)	910 (68.1%)
	Unknown	20 (28.6%)	355 (26.5%)
Deprivation Index (N, %)	Unknown	*	185 (13.7%)
	1-2	30 (46%)	410 (35.8%)
	3-4	10 (17.4%)	205 (17.9%)
	5-6	*	210 (18.4%)
	7-8	10 (17.4%)	200 (17.2%)
	9-10	*	120 (10.6%)
Outcome (N, %)	Death	45 (65.7%)	1,110 (83.3%)

For peer review only - http://bmjopen.bmj.com/site/about/guidelines.xhtml

	Deaths due to COVID (including after 30 days)	20 (27.2%)	520 (39%)
	Organ support (within 30 days)	35 (47.1%)	300 (22.6%)
**Hospitalisation (N, %)**	ED attendance	45 (65.7%)	925 (69.2%)
	Inpatient admission	50 (72.9%)	1,035 (77.6%)
**Confirmed Hospital Diagnosis of COVID (N, %)** **	In ED or as inpatient at 30 days	25 (34.3%)	570 (42.7%)
**Time to Primary Outcome from index contact- up to and including (N, %)**	72 hours	25 (37.1%)	445 (33.5%)
	7 days	45 (62.8%)	730 (54.9%)

Table 4 compares the characteristics of patients without the primary outcome (death or organ support) for whom an ambulance was dispatched and the patient was not transferred to hospital (False Positives), or no ambulance was dispatched (True Negatives). 33.4% of the cohort were false positives and Supplementary Material 3 presents the results of multivariable modelling to identify factors associated with false postivie triage. Risk of false positive was strongly associated with chronic respiratory disease (OR 1.35 95% CI 1.13 to 1.60) and reduced in smokers (OR 0.86, 95% CI 0.75 to 0.99). Increasing age, deprivation and female sex were also associated with risk of false positive triage.

Table 4: False positive compared to true negatives

Population Characteristic	Level	False Positive (Ambulance dispatched, not conveyed to hospital and no primary outcome 30 days) N= 4,230	True Negative (No ambulance dispatched and no primary outcome 30 days) N= 1,930
**Age (Years)**	Median (IQR)<br>Mean	66 (46-81)<br>62.9	37 (28-53)<br>41.6
**Sex (N, %)**	Male	1,920 (45.4%)	1000 (51.7%)
**Comorbidity (N, %)**	Cardiovascular Disease	235 (5.5%)	30 (1.6%)
	Chronic Resp. Disease	1,280 (30.3%)	380 (19.5%)
	Diabetes	600 (14.2%)	155 (7.8%
	Hypertension	1,480 (35%)	255(13.1%)
	Immunosuppression (including steroid use)	825 (19.5%)	180 (9.4%)
	Active Malignancy	140 (3.3%)	20 (0.9%)
	Obesity	700 (16.5%)	260 (13.4%)
	Pregnant	50 (1.2%)	40 (2.2%)
	Renal Impairment	110 (2.6%)	15 (0.8%)

For peer review only - http://bmjopen.bmj.com/site/about/guidelines.xhtml

	Stroke	90 (2.1%)	15 (0.7%)
**Smoking Status (N, %)**	Smoker	1,570 (37.1%)	640 (33.2%)
**Number of Drugs Used (N, %)**	0	1,040 (24.6%)	955 (49.3%)
	1-5	2,075 (49%)	805 (41.7%)
	6-10	970 (22.9%)	150 (7.6%)
	11 or more	150 (3.5%)	25 (1.4%)
**Clinical Frailty Scale (N, %)**	Unknown	1,520 (35.9%)	150 (7.6%)
	Aged<65	2,030 (48%)	1,735 (89.8%)
	1-3	55 (1.3%)	*
	3-6	245 (5.7%)	25 (1.2%)
	6-9	385 (9.1%)	25 (1.2%)
**Ethnicity (N, %)**	Asian or Asian British	250 (5.9%)	180 (9.2%)
	Black or Black British	60 (1.4%)	50 (2.5%)
	Mixed	35 (0.9%)	30 (1.4%)
	Other Ethnic Groups	50 (1.2%)	65 (3.3%)
	White	3,080 (72.7%)	1,210 (62.7%)
	Unknown	760 (17.9%)	405 (20.9%)
**Deprivation Index (N, %)**	Unknown	420 (10%)	130 (6.6%)
	1-2	1,470 (38.6%)	925 (51.3%)
	3-4	720 (18.9%)	320 (17.7%)
	5-6	655 (17.2%)	245 (13.5%)
	7-8	560 (14.7%)	190 (10.5%)
	9-10	405 (10.6%)	125 (6.9%)

Discussion

Summary

Our study showed that callers with suspected COVID-19 who made an EMS call had a high rate of adverse outcomes (death or organ support) (11.1%, 95% CI: 10.5 to 11.7%). This is around four times greater than the adverse outcome rate in callers who contacted the NHS 111 telephone service and half that seen in an ED population with suspected COVID-19.[18, 21] Ambulances were dispatched to the majority of callers (84.2%) and the decision to dispatch an ambulance achieved a sensitivity of 95%(95% CI: 93.7 to 96.1%) for the primary outcome (death or organ support). Callers for whom an ambulance was not dispatched had a 3.5% or around 1/29 (NPP 96.5% 95% CI: 95.6 to 97.2%) risk of the primary composite adverse outcome. This equated to 70 callers who died or required organ support up to 30 days from first call, not initially being dispatched an ambulance. Our evaluation cannot account for instances where ambulance dispatch was not clinically appropriate despite a high-risk of significant adverse outcomes (e.g. transfer of care to community services for palliative care).

The cost of the high sensitivity achieved by dispatching ambulances to 84% of callers was a low specificity to the primary outcome (death or organ support) (17.2% 95% CI: 16.5% to 17.9%). In total,

For peer review only - http://bmjopen.bmj.com/site/about/guidelines.xhtml

33.4% of the cohort had an ambulance dispatched, and were not subsequently conveyed to hospital and nor did they experience the primary outcome. We used multivariable analysis to identify predictors of false negative and false positive triage. The findings need cautious interpretation, given the limited information available during telephone triage, but suggest that some co-morbidities (such as chronic respiratory disease) and increasing age may be over-estimated as predictors of adverse outcome.

Strengths and limitations

Although specific EMS telephone triage protocols have been introduced for patients with suspected COVID-19, this appears to be the first evaluation of triage accuracy.[4] Our study used a large cohort of patients identified from routinely collected EMS records and linked this to nationally collected, patient-level healthcare data to provide robust clinical outcomes. We have assessed performance in a cohort of patients with suspected infection which, in the absence of accurate universally available rapid COVID-19 diagnostic tests, reflects the population who must be clinically triaged by urgent and emergency care services.

We have evaluated the performance EMS telephone triage for patients with suspected COVID-19 implemented by the Yorkshire Ambulance Service NHS Trust. Although use of the Card 36 protocol was recommended nationally, there was variation in implementation between different ambulance services.[4] Our study used data from the first wave of the pandemic and it was not until later waves that some ambulance services came under significant pressures due to increased demand.[1] Differences in EMS telephone triage demand and population characteristics of callers with suspected COVID-19 in later waves of the pandemic may affect the estimated accuracy of EMS decision to dispatch an ambulance. The implementation of a senior clinical support model within the call centre, and the influence of this secondary triage both of incoming calls and to support on-scene ambulance decision making was not quantified in this study. It was not possible to identify within the dataset which of the cohort were reviewed by a senior clinician at the initial call.

Our study used routinely collected data and consequently there were high rates of missing data for some variables, such as ethnicity and frailty. This prevented inclusion of these and non-routinely collected variables in analysis. We have assumed that if comorbidities or medication use was not recorded, they were not present. We also assumed in multivariable analysis, patients under the age of 65 had a functional level equivalent to a mild frailty category (CFS 1-3). The mechanism of how data are collected and recorded in the routine data sets used means that there may be bias in the classification of patients. We have previously identified this for the obesity variable where the estimated prevalence of obesity in our cohort is 15% (half that reported in the national health

For peer review only - http://bmjopen.bmj.com/site/about/guidelines.xhtml

survey) and an implausible protective association with adverse outcomes was observed.[17, 18] As weight is not comprehensively and consistently measured by GPs, estimated statistical effects may reflect unknown characteristics associated with a measurement being taken, rather than the variable itself.

Implications

EMS telephone triage of ambulance dispatch achieved a higher sensitivity for adverse outcomes (death or organ support) than triage methods used for patient acuity in the ED and NHS-111 COVID-19 assessment pathways.[22, 23] Our cohort had a baseline risk of 11.1% of the primary outcome (death or organ support). The EMS telephone triage system selected those with a lower, 3.5% risk of the primary outcome (death or organ support), not to have an ambulance dispatched. Given the high baseline risk, an alternative would have been to dispatch an ambulance to every caller. This would have led to around 2000 more ambulance being dispatched within the time-period (an 18.8% increase). The acceptable risk of deterioration following telephone triage is subjective and significant variation in risk tolerance between clinicians and public representatives has been demonstrated.[24] When discharging patients from the ED with chest pain, an acceptable risk of a subsequent major adverse cardiovascular events has been found to be 1% or less for the majority of ED clinicians.[25] The risk of false negative triage (3.5%) in our cohort may therefore be unacceptably high for some clinicians. However, this is within the context of a face-to-face assessment with the availability of hospital investigations where greater accuracy may be expected.

Even using EMS telephone triage of ambulance dispatch, around a third of ambulances were dispatched to callers who were not subsequently conveyed to hospital and did not experience the primary outcome. In later waves of the pandemic, EMS providers in the UK experienced significant increases in demand with several ambulance services declaring major incidents.[8, 26, 27] The accuracy of triage observed in our cohort may be the best that realistically can be achieved given the limited information available with telephone triage. However, any measures which can increase accuracy of EMS telephone triage are greatly needed.

Our exploratory multivariable analysis indicates that older age and presence of pre-existing respiratory disease were associated with a higher rate of false positive triage among those who did not have an adverse event (death or organ support), and therefore may be overestimated in the triage process as predictors of adverse outcomes. Being aged 65 years or over is included as a specific high-risk factor in the Card 36 pandemic triage tool.[28] Use of a higher age threshold, or age in conjunction with variables related to performance status, could improve the specificity of EMS ambulance dispatch to significant adverse outcomes.[13] Vaccination against COVID-19 or previous

For peer review only - http://bmjopen.bmj.com/site/about/guidelines.xhtml

infection may also act to reduce the risk of serious adverse outcomes in suspected infection and these factors are not included in Card 36 assessment.[29]

Limited information is available during EMS telephone triage determining whether ambulance dispatch is required. The Card 36 pandemic triage tool used for this decision making in patients with suspected COVID-19 is consensus based. Empirical research assessing the predictive effect of all available variables is required if accuracy of triage, especially in terms of current over-triage and risk of false-negative triage, is to be improved. Given the limitations of the available routine data we have identified in this study, this is likely to require robust prospective data collection to empirically develop and validate more accurate triage tools. The use of trained, non-clinical call handlers for ambulance dispatch contrasts with other telephone triage services.[30] Other models of assessment could improve accuracy and also require evaluation. The current high levels of demand that ambulance services are experiencing mean that even small gains in accuracy could have a large positive effect on both safety and managing increasing demand.

Conclusion

EMS telephone triage of need for ambulance dispatch in the first wave of the pandemic identified a lower risk population to whom ambulances were not dispatched. They constituted 16% of the cohort and around 2000 more ambulances would have been dispatched if these callers received an EMS response. However, patients to whom an ambulance was not dispatched had a clinically significant risk of death or requiring organ support. As the pandemic has developed into later waves, ambulance services in the UK have come under significant sustained pressure due to increased demand. Research to improve the accuracy of EMS telephone triage, especially in terms of safety and specificity, is needed. Due to the limitations of available routinely collected data this is likely to require robust prospective data collection.

Competing Interests

No competing financial interests exist.

Funding

CM is a National Institute for Health Research (NIHR) Clinical Lecturer in Emergency Medicine (Grant Number Not Applicable/NA). The PRIEST study was funded by the UK National Institute for Health Research, Health Technology Assessment (HTA) programme (project reference 11/46/07). This publication presents independent research funded by the National Institute for Health Research and University of Sheffield. The views expressed are those of the author(s) and not necessarily those of the University of Sheffield, the NHS, the NIHR or the Department of Health and Social Care.

For peer review only - http://bmjopen.bmj.com/site/about/guidelines.xhtml

Authors' contributions

The idea for the study was conceived by SG, JT, TS, FB, PB, and CM. Data processing and linkage was completed by TS and RC. The analyses were completed by CM and MH with specialist statistical advice from SG, JT, LS and PB. CM, TS, MH, RC, PB, JT, RP, BT, LS, FB, KB, FH, SM, JP, and SG contributed to interpretation of results, read, and approved the final manuscript.

Data sharing:

The data used for this study are subject to data sharing agreements with NHS Digital and Yorkshire Ambulance Service which prohibits further sharing of individual level data. The data sets used are obtainable from these organisations subject to necessary authorisations and approvals.

Figures:

Figure 1: STROBE flow diagram of selection of study population

For peer review only

References:

1. https://www.hsj.co.uk/emergency-care/thousands-of-londons-999-calls-answered-by-other-regions/7029302.article.
2. https://www.nationalleadership.gov.uk/supporting-the-nhs-in-its-hour-of-need/london-ambulance-service/.
3. Jensen T, Holgersen MG, Jespersen MS, et al. Strategies to Handle Increased Demand in the COVID-19 Crisis: A Coronavirus EMS Support Track and a Web-Based Self-Triage System. *Prehospital Emergency Care.* 2021;25:28-38.
4. Snooks H, Watkins AJ, Bell F, et al. Call volume, triage outcomes, and protocols during the first wave of the COVID-19 pandemic in the United Kingdom: Results of a national survey. *Journal of the American College of Emergency Physicians Open.* 2021;2:e12492.

For peer review only - http://bmjopen.bmj.com/site/about/guidelines.xhtml

5. Ferron R, Agarwal G, Cooper R, Munkley D. The effect of COVID-19 on emergency medical service call volumes and patient acuity: a cross-sectional study in Niagara, Ontario. *BMC Emergency Medicine.* 2021;21:39.
6. Charlton K, Limmer M, Moore H. Incidence of emergency calls and out-of-hospital cardiac arrest deaths during the COVID-19 pandemic: findings from a cross-sectional study in a UK ambulance service. *Emergency Medicine Journal.* 2021;38:446.
7. Blackwell TH, Kaufman JS. Response time effectiveness: comparison of response time and survival in an urban emergency medical services system. *Academic Emergency Medicine.* 2002;9:288-295.
8. https://www.london.gov.uk/press-releases/mayoral/hospitals-at-risk-of-being-overwhelmed-in-capital.
9. https://www.gov.uk/government/publications/covid-19-guidance-for-ambulance-trusts/covid-19-guidance-for-ambulance-trusts.
10. https://www.emergencydispatch.org/in-the-news/covid-19-communications/a332c5d1-0dbf-4354-9a64-8d149f75f15d.
11. Tankel JW, Ratcliffe D, Smith M, et al. Consequences of the emergency response to COVID-19: a whole health care system review in a single city in the United Kingdom. *BMC Emergency Medicine.* 2021;21:55.
12. https://digital.nhs.uk/services/national-data-opt-out.
13. Goodacre S, Thomas B, Sutton L, et al. Derivation and validation of a clinical severity score for acutely ill adults with suspected COVID-19: The PRIEST observational cohort study. *PLoS One.* 2021;16:e0245840.
14. Campbell MJ, Jacques RM, Fotheringham J, Maheswaran R, Nicholl J. Developing a summary hospital mortality index: retrospective analysis in English hospitals over five years. *BMJ.* 2012;344:e1001.
15. Street A, Maynou L, Gilbert T, Stone T, Mason S, Conroy S. The use of linked routine data to optimise calculation of the Hospital Frailty Risk Score on the basis of previous hospital admissions: a retrospective observational cohort study. *The Lancet Healthy Longevity.* 2021;2:e154-e162.
16. Rockwood K, Song X, MacKnight C, et al. A global clinical measure of fitness and frailty in elderly people. *CMAJ.* 2005;173:489-495.
17. https://digital.nhs.uk/data-and-information/publications/statistical/health-survey-for-england/2019.
18. Marincowitz C, Stone T, Bath P, et al. Accuracy of telephone triage for predicting adverse outcome in suspected COVID-19: An observational cohort study. *medRxiv.* 2021:2021.2006.2024.21259441.
19. Hirst E, Irving A, Goodacre S. Patient and public involvement in emergency care research. *Emergency Medicine Journal.* 2016;33:665-670.
20. https://digital.nhs.uk/binaries/content/assets/website-assets/publications/publications-admin-pages/related-documents/disclosure-control-procedure.pdf.
21. Goodacre S, Thomas B, Lee E, et al. Characterisation of 22445 patients attending UK emergency departments with suspected COVID-19 infection: Observational cohort study. *PLOS ONE.* 2020;15:e0240206.
22. Zachariasse JM, van der Hagen V, Seiger N, Mackway-Jones K, van Veen M, Moll HA. Performance of triage systems in emergency care: a systematic review and meta-analysis. *BMJ Open.* 2019;9:e026471.
23. Thomas B, Goodacre S, Lee E, et al. Prognostic accuracy of emergency department triage tools for adults with suspected COVID-19: the PRIEST observational cohort study. *Emergency Medicine Journal.* 2021:emermed-2020-210783.

For peer review only - http://bmjopen.bmj.com/site/about/guidelines.xhtml

24. O'Keeffe ST. A cross-sectional study of doctors', managers' and public representatives' views regarding acceptable level of risk in discharges from the emergency department. *Qjm.* 2015;108:533-538.

25. Than M, Herbert M, Flaws D, et al. What is an acceptable risk of major adverse cardiac event in chest pain patients soon after discharge from the Emergency Department?: A clinical survey. *International Journal of Cardiology.* 2013;166:752-754.

26. https://www.yorkpress.co.uk/news/19012031.yorkshire-ambulance-service-declares-major-incident---report/.

27. https://www.reuters.com/article/us-health-coronavirus-britain-manchester-idUKKBN27I2HK.

28. https://cdn.emergencydispatch.org/iaed/pdf/NAE-Protocol-36-Flu-v13_3.pdf.

29. Abbasi J. Study Suggests Lasting Immunity After COVID-19, With a Big Boost From Vaccination. *JAMA.* 2021;326:376-377.

30. van Ierland Y, van Veen M, Huibers L, Giesen P, Moll HA. Validity of telephone and physical triage in emergency care: The Netherlands Triage System. *Family Practice.* 2011;28:334-341.

For peer review only

For peer review only - http://bmjopen.bmj.com/site/about/guidelines.xhtml

Figure 1: STROBE flow diagram of study population selection (Created by the authors)

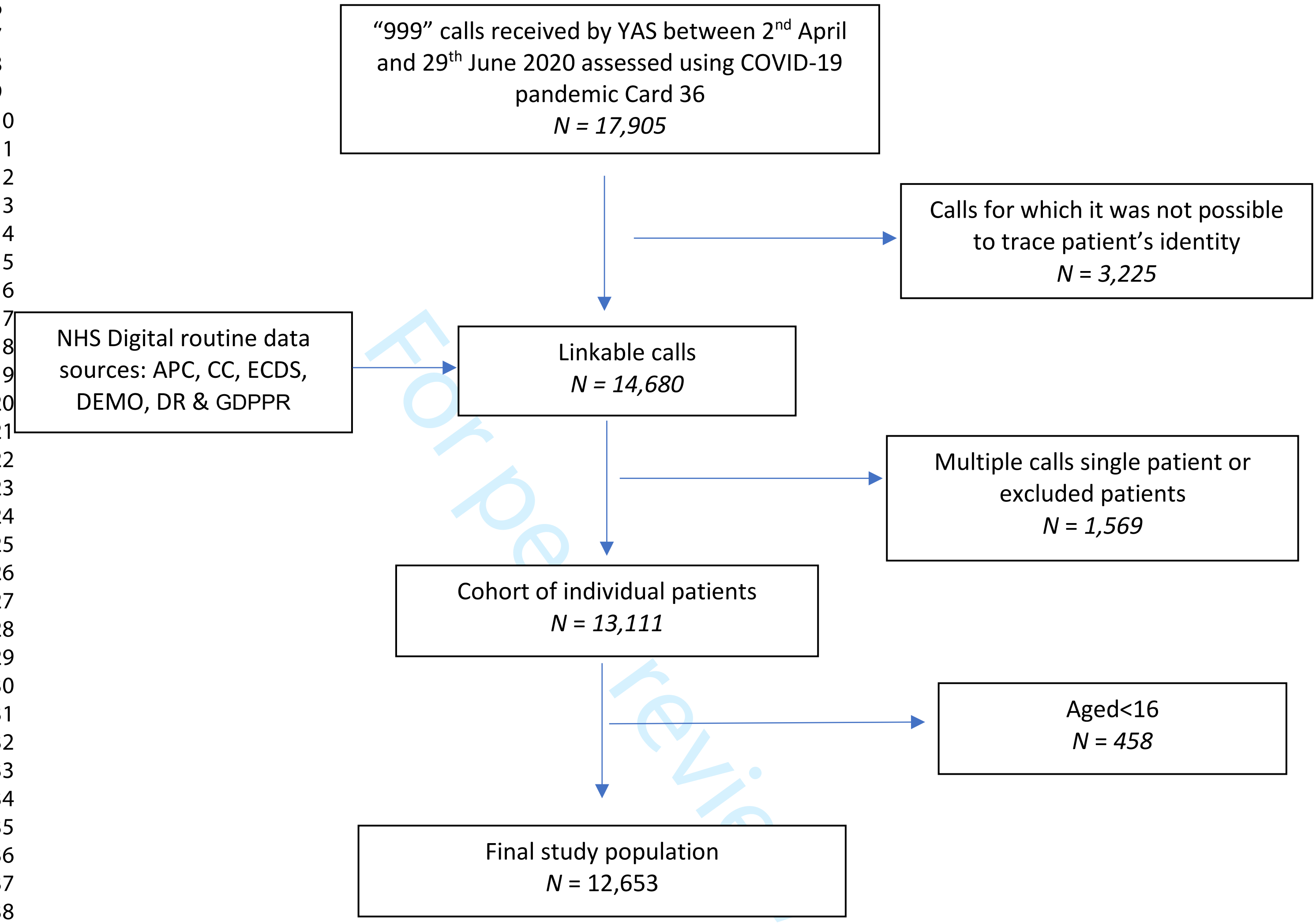

For peer review only

Supplementary Material 1: Summary of AMPDS pandemic Card 36 (Created by the authors) (full details available: https://cdn.emergencydispatch.org/iaed/pdf/resource-library/public%20protocols/pub-protocol%2036/NAE%20CC%2036.pdf)

Questions	Outcome
1) What is the most prominent complaint?	**Delta Category (immediate response)**<br>**Category 1/2 UK Ambulance service response**<br>1) INEFFECTIVE BREATHING with flu-like symptoms<br>2) DIFFICULTY SPEAKING BETWEEN BREATHS with flu-like symptoms<br>3) Not alert with flu-like symptoms<br>4) CHANGING COLOR with flu-like symptoms
If breathing a)Do they have difficulty speaking between breathes?<br>b) Describe their breathing<br>c)Did they have any flu symptoms prior to this?	
If Chest pain a) Are they 35 and over?<br>b) Have they had a heart attack or angina previously	
2) Are they completely alert?	
3) Are they changing colour?	
4) Are they having chills or sweats?	**Charlie Category (Ambulance when able)**<br>**Category 3/4 UK Ambulance service response**<br>1) Abnormal breathing with single flu-like symptom or Asthma/COPD<br>2) Abnormal breathing with multiple flu-like symptoms<br>3) Chest pain/discomfort ≥35 with single flu-like symptom<br>4) Chest pain/discomfort ≥35 with multiple flu-like symptoms<br>5) High Risk Factor
5) Are they vomiting?	
6)Do they have a new cough that recently started?	
7)Do they have a sore throat?	
8)Do they have unusual total body aches?	
9)Do they have a fever?	**Alpha Category (No ambulance dispatched)**<br>1) Chest pain/discomfort < 35 with single flu-like symptom<br>2) Chest pain/discomfort < 35 with multiple flu-like symptoms<br>3) Flu-like symptoms only
10)Do they have a runny or snotty nose?	
11)Do they have diarrhoea?	
12)Do they have a headache?	
13)Do they have any high-risk conditions?<br>• ≤ 5 years old (not COVID-19)<br>• ≥ 65 years old<br>• Blood disorders<br>• Diabetes<br>• Kidney and liver diseases/disorders<br>• Neurological diseases<br>• Pregnancy (up to 2 weeks after delivery)<br>• Sickle cell disease (sickle cell anaemia)<br>• Weakened immune system	

For peer re

Supplementary Material 2: Multi-variable model predicting false negatives (Created by the authors)

Population Characteristic	Level	Odds ratio (95% CI) N= 1215
**Age (Years)**	1-year increase	0.95 (0.94 to 0.97)
**Sex**	Female	1.89 (1.09 to 3.26)
**Comorbidity**	Cardiovascular Disease	0.73 (0.21 to 2.56)
	Chronic Resp. Disease	0.50 (0.23 to 1.09)
	Diabetes	1.19 (0.60 to 2.38)
	Hypertension	0.75 (0.39 to 1.46)
	Immunosuppression (including steroid use)	0.73 (0.35 to 1.53)
	Active Malignancy	0.12 (0.02 to 0.92)
	Obesity	Not included
	Renal Impairment	2.22 (0.71 to 6.96)
	Smoker	0.96 (0.52 to 1.80)
	Stroke	1.93 (0.42 to 8.80)
**Number of Drugs Used**	0	Reference
	1-5	0.98 (0.45 to 2.15)
	6-10	1.56 (0.55 to 4.44)
	11 or more	0.76 (0.08 to 7.42)
**Deprivation Index**	1-2	Reference
	3-4	0.98 (0.47 to 2.08)
	5-6	0.54 (0.21 to 1.36)
	7-8	1.37 (0.64 to 2.97)
	9-10	0.99 (0.38 to 2.59)

For peer review only

Supplementary Material 3: Multi-variable model predicting false positives

Population Characteristic	Level	Odds ratio (95% CI) N= 5, 615
**Age (Years)**	1-year increase	1.05 (1.04 to 1.05)
**Sex**	Female	1.31 (1.15 to 1.49)
**Comorbidity**	Cardiovascular Disease	1.38 (0.85 to 2.24)
	Chronic Resp. Disease	1.35 (1.13 to 1.60)
	Diabetes	0.85 (0.67 to 1.09)
	Hypertension	1.03 (0.84 to 1.26)
	Immunosuppression (including steroid use)	1.02 (0.82 to 1.26)
	Active Malignancy	1.25 (0.72 to 2.18)
	Renal Impairment	0.80 (0.44 to 1.44)
	Smoker	0.86 (0.75 to 0.99)
	Stroke	1.17 (0.59 to 2.31)
**Number of Drugs Used**	0	Reference
	1-5	0.99 (0.84 to 1.16)
	6-10	1.22 (0.90 to 1.66)
	11 or more	1.13 (0.62 to 2.06)
**Deprivation Index**	1-2	Reference
	3-4	1.21 (1.02 to 1.44)
	5-6	1.23 (1.01 to 1.48)
	7-8	1.29 (1.05 to 1.59)
	9-10	1.32 (1.04 to 1.69)

For peer review only

The RECORD statement – checklist of items, extended from the STROBE statement, that should be reported in observational studies using routinely collected health data.

	Item No.	STROBE items	Location in manuscript where items are reported	RECORD items	Location in manuscript where items are reported
**Title and abstract**					
	1	(a) Indicate the study's design with a commonly used term in the title or the abstract (b) Provide in the abstract an informative and balanced summary of what was done and what was found	Pages 1-2	RECORD 1.1: The type of data used should be specified in the title or abstract. When possible, the name of the databases used should be included.<br><br>RECORD 1.2: If applicable, the geographic region and timeframe within which the study took place should be reported in the title or abstract.<br><br>RECORD 1.3: If linkage between databases was conducted for the study, this should be clearly stated in the title or abstract.	Page 2 (abstract)
**Introduction**					
Background rationale	2	Explain the scientific background and rationale for the investigation being reported	Page 3		
Objectives	3	State specific objectives, including any prespecified hypotheses	Page 3		
**Methods**					
Study Design	4	Present key elements of study design early in the paper	Page 4		
Setting	5	Describe the setting, locations, and relevant dates, including periods of recruitment, exposure, follow-up, and data collection	Page 4		

For peer review only

Participants	6	*(a) Cohort study* - Give the eligibility criteria, and the sources and methods of selection of participants. Describe methods of follow-up<br>*Case-control study* - Give the eligibility criteria, and the sources and methods of case ascertainment and control selection. Give the rationale for the choice of cases and controls<br>*Cross-sectional study* - Give the eligibility criteria, and the sources and methods of selection of participants<br><br>*(b) Cohort study* - For matched studies, give matching criteria and number of exposed and unexposed<br>*Case-control study* - For matched studies, give matching criteria and the number of controls per case	Page 4,5	RECORD 6.1: The methods of study population selection (such as codes or algorithms used to identify subjects) should be listed in detail. If this is not possible, an explanation should be provided.<br><br>RECORD 6.2: Any validation studies of the codes or algorithms used to select the population should be referenced. If validation was conducted for this study and not published elsewhere, detailed methods and results should be provided.<br><br>RECORD 6.3: If the study involved linkage of databases, consider use of a flow diagram or other graphical display to demonstrate the data linkage process, including the number of individuals with linked data at each stage.	Page 4,5<br>Figure 1
Variables	7	Clearly define all outcomes, exposures, predictors, potential confounders, and effect modifiers. Give diagnostic criteria, if applicable.	Page 5,6	RECORD 7.1: A complete list of codes and algorithms used to classify exposures, outcomes, confounders, and effect modifiers should be provided. If these cannot be reported, an explanation should be provided.	
Data sources/ measurement	8	For each variable of interest, give sources of data and details of methods of assessment (measurement).<br>Describe comparability of assessment methods if there is more than one group	Page 5,6		

For peer review only

Bias	9	Describe any efforts to address potential sources of bias	Page 5-6		
Study size	10	Explain how the study size was arrived at	Pages 4,5 Figure 1		
Quantitative variables	11	Explain how quantitative variables were handled in the analyses. If applicable, describe which groupings were chosen, and why	Pages 5,6		
Statistical methods	12	(a) Describe all statistical methods, including those used to control for confounding<br>(b) Describe any methods used to examine subgroups and interactions<br>(c) Explain how missing data were addressed<br>(d) *Cohort study* - If applicable, explain how loss to follow-up was addressed<br>*Case-control study* - If applicable, explain how matching of cases and controls was addressed<br>*Cross-sectional study* - If applicable, describe analytical methods taking account of sampling strategy<br>(e) Describe any sensitivity analyses	Pages 5,6		
Data access and cleaning methods		..	Page 4	RECORD 12.1: Authors should describe the extent to which the investigators had access to the database population used to create the study population.	Page 4

For peer review only

				RECORD 12.2: Authors should provide information on the data cleaning methods used in the study.	
Linkage		..	Page 4	RECORD 12.3: State whether the study included person-level, institutional-level, or other data linkage across two or more databases. The methods of linkage and methods of linkage quality evaluation should be provided.	Figure 1
**Results**					
Participants	13	(a) Report the numbers of individuals at each stage of the study (*e.g.*, numbers potentially eligible, examined for eligibility, confirmed eligible, included in the study, completing follow-up, and analysed)<br>(b) Give reasons for non-participation at each stage.<br>(c) Consider use of a flow diagram	Figure 1	RECORD 13.1: Describe in detail the selection of the persons included in the study (*i.e.,* study population selection) including filtering based on data quality, data availability and linkage. The selection of included persons can be described in the text and/or by means of the study flow diagram.	Figure 1
Descriptive data	14	(a) Give characteristics of study participants (*e.g.*, demographic, clinical, social) and information on exposures and potential confounders<br>(b) Indicate the number of participants with missing data for each variable of interest<br>(c) *Cohort study* - summarise follow-up time (*e.g.*, average and total amount)	Table 1		
Outcome data	15	*Cohort study* - Report numbers of outcome events or summary measures over time<br>*Case-control study* - Report numbers in each exposure	Page 6-8		

For peer review only

		category, or summary measures of exposure<br>*Cross-sectional study* - Report numbers of outcome events or summary measures			
Main results	16	(a) Give unadjusted estimates and, if applicable, confounder-adjusted estimates and their precision (e.g., 95% confidence interval). Make clear which confounders were adjusted for and why they were included<br>(b) Report category boundaries when continuous variables were categorized<br>(c) If relevant, consider translating estimates of relative risk into absolute risk for a meaningful time period	Pages 8-11		
Other analyses	17	Report other analyses done—e.g., analyses of subgroups and interactions, and sensitivity analyses	Pages 8-11		
**Discussion**					
Key results	18	Summarise key results with reference to study objectives	Pages 12		
Limitations	19	Discuss limitations of the study, taking into account sources of potential bias or imprecision. Discuss both direction and magnitude of any potential bias	Pages 12-13	RECORD 19.1: Discuss the implications of using data that were not created or collected to answer the specific research question(s). Include discussion of misclassification bias, unmeasured confounding, missing data, and changing eligibility over time, as they pertain to the study being reported.	Pages 12-13
Interpretation	20	Give a cautious overall interpretation of results considering objectives,	Pages 13-14		

For peer review only - http://bmjopen.bmj.com/site/about/guidelines.xhtml

		limitations, multiplicity of analyses, results from similar studies, and other relevant evidence			
Generalisability	21	Discuss the generalisability (external validity) of the study results	Pages 12-14		
**Other Information**					
Funding	22	Give the source of funding and the role of the funders for the present study and, if applicable, for the original study on which the present article is based	15		
Accessibility of protocol, raw data, and programming code		..	15	RECORD 22.1: Authors should provide information on how to access any supplemental information such as the study protocol, raw data, or programming code.	15

*Reference: Benchimol EI, Smeeth L, Guttmann A, Harron K, Moher D, Petersen I, Sørensen HT, von Elm E, Langan SM, the RECORD Working Committee. The REporting of studies Conducted using Observational Routinely-collected health Data (RECORD) Statement. *PLoS Medicine* 2015; in press.

*Checklist is protected under Creative Commons Attribution (CC BY) license.

For peer review only
